# Co-Aggregation of TDP-43 with Other Pathogenic Proteins and Their Co-Pathologies in Neurodegenerative Diseases

**DOI:** 10.3390/ijms252212380

**Published:** 2024-11-18

**Authors:** Lei-Lei Jiang, Xiang-Le Zhang, Hong-Yu Hu

**Affiliations:** 1 Key Laboratory of RNA Innovation, Science and Engineering, Shanghai Institute of Biochemistry and Cell Biology, Center for Excellence in Molecular Cell Science, Chinese Academy of Sciences, Shanghai 200031, China; jiangleilei@sibcb.ac.cn (L.-L.J.); zhangxiangle2020@sibcb.ac.cn (X.-L.Z.); 2 University of Chinese Academy of Sciences, Beijing 100049, China

**Keywords:** TDP-43 pathology, co-aggregation, pathogenic protein, co-pathology, neurodegenerative disease

## Abstract

Pathological aggregation of a specific protein into insoluble aggregates is a common hallmark of various neurodegenerative diseases (NDDs). In the earlier literature, each NDD is characterized by the aggregation of one or two pathogenic proteins, which can serve as disease-specific biomarkers. The aggregation of these specific proteins is thought to be a major cause of or deleterious result in most NDDs. However, accumulating evidence shows that a pathogenic protein can interact and co-aggregate with other pathogenic proteins in different NDDs, thereby contributing to disease onset and progression synergistically. During the past years, more than one type of NDD has been found to co-exist in some individuals, which may increase the complexity and pathogenicity of these diseases. This article reviews and discusses the biochemical characteristics and molecular mechanisms underlying the co-aggregation and co-pathologies associated with TDP-43 pathology. The TDP-43 aggregates, as a hallmark of amyotrophic lateral sclerosis (ALS) and frontotemporal lobar degeneration (FTLD), can often be detected in other NDDs, such as Alzheimer’s disease (AD), Parkinson’s disease (PD), Huntington’s disease (HD) and spinocerebellar ataxia type 2 (SCA2). In many cases, TDP-43 is shown to interact and co-aggregate with multiple pathogenic proteins in vitro and in vivo. Furthermore, the co-occurrence and co-aggregation of TDP-43 with other pathogenic proteins have important consequences that may aggravate the diseases. Thus, the current viewpoint that the co-aggregation of TDP-43 with other pathogenic proteins in NDDs and their relevance to disease progression may gain insights into the patho-mechanisms and therapeutic potential of various NDDs.

## 1. Introduction

The accumulation of protein aggregates and the formation of inclusion bodies are closely associated with the pathogenesis and progression of neurodegenerative diseases (NDDs) [1,2,3,4,5], such as Alzheimer’s disease (AD), Parkinson’s disease (PD), Huntington’s disease (HD), spinocerebellar ataxia type 2 (SCA2) and amyotrophic lateral sclerosis (ALS). So far, more than 50 pathogenic proteins or peptides prone to aggregation and a large number of pathologies related to protein aggregation have been discovered and investigated in detail [2,6].

According to the traditional viewpoint, the aggregation of one or two specific proteins is regarded as a characteristic of a disease, and severing is the biomarker [2,3,4,5]; for example, as seen in amyloid β (Aβ) and the Tau protein (Tau) for AD [7,8,9], α-synuclein (α-Syn) for PD [10,11,12], huntingtin (Htt) for HD [13,14] and transactive response DNA-binding protein 43 (TDP-43) for ALS and frontotemporal lobar degeneration (FTLD) [15,16]. The aggregates formed by pathogenic proteins have been reported to sequester other cellular factors and cause dysfunction and impairment of the sequestered components [17,18,19], which is implicated in the pathologies of NDDs [20,21]. Intriguingly, in many cases, the co-aggregation of multiple pathogenic proteins can also occur in the same cell, contributing to clinical presentation and disease progression, hereto termed co-pathology, implying the complexity of protein aggregation and potential interactions among the pathogenic proteins [22,23,24,25,26,27,28]. In fact, these pathogenic proteins can interact and co-aggregate with each other in vitro and in vivo, influencing the clinical and neuropathological features of NDDs synergistically. For instance, Tau can interact and co-aggregate with α-Syn, and this association increases aggregation and neurotoxicity in cell [29], *Drosophila* [30], mouse [31] and in vitro models [32,33], which are essential for the development and spreading of neurodegeneration. Even then, there is an overlap among Tau, α-Syn and Aβ in multiple pathologies, which, combined, accelerate the neurodegeneration and disease progression [34,35,36]. Further, mutant Htt (mHtt) can co-aggregate with α-Syn and promote their aggregation and synergistic neurotoxicity in in vitro and in vivo models [37,38,39,40,41], which consequently influences the disease progression and clinical manifestation. Likewise, mHtt has also been shown to co-aggregate with Tau to modulate their aggregation properties, leading to worsening neurodegeneration and behavioral impairments [40,42,43,44,45]. Of note, different polyglutamine (polyQ) proteins can co-aggregate with and sequester each other by their polyQ-tract interactions and impair their cellular proteostasis [46,47,48]. At the same time, numerous studies have shown that some individuals exhibit a concurrence of more than one NDD and confirmed the observation in the post-mortem brains of patients, which may influence the diseases with or without clinical presentation [27,49,50,51,52]. In addition, the prion-like cell-to-cell transmission of the pathogenic protein aggregates is also involved in the onset and progression of various NDDs [53,54,55].

Altogether, these findings reveal the co-existence of pathogenic protein aggregates and their co-pathologies in disease progression, providing supporting evidence for a more common mechanism underlying different NDDs. That is, the aggregation of one protein may trigger the aggregation of other pathogenic proteins through intermolecular interaction, co-aggregation and mutual sequestration. Such accumulation of the pathogenic proteins into inclusions leads to the loss of essential function or gain of toxic function of the sequestered proteins, which may influence the biological processes or pathways, eventually aggravate the aggregation-related protein pathologies (proteinopathies) and accelerate the disease progression [23,24,26,27,56]. Therefore, the co-aggregation of pathogenic proteins plays an important role in the co-pathologies of NDDs.

As the TDP-43 aggregates are found in ALS, FTLD and many other NDDs referred to as TDP-43 co-pathologies, elucidating the mechanism of pathogenic protein co-aggregation associated with TDP-43 becomes more important in understanding the co-pathologies of NDDs related to TDP-43 pathology. In this review article, we will summarize and discuss the characteristic features of TDP-43 pathology, gain insights into the co-aggregation of TDP-43 with other pathogenic proteins and, particularly, highlight their potential mechanism and relevance to the pathogenesis of NDDs. This will be beneficial to our better understanding of the TDP-43-related co-aggregation and co-pathologies, which may have significant physiological and pathological impacts on the mechanisms of various NDDs.

## 2. TDP-43 Protein and TDP-43 Co-Pathologies

### 2.1. Physiological and Pathological TDP-43

TDP-43 is a highly conserved and ubiquitously expressed RNA/DNA-binding protein, belonging to the hnRNP family [57,58]. The structure of TDP-43 is composed of an N-terminal domain (NTD), two RNA recognition motifs (RRMs), a nuclear localization signal (NLS), a nuclear export signal (NES) and a C-terminal domain (CTD) [57,59]. The protein is predominantly localized in the nucleus, but it can also shuttle between the cytoplasm and nucleus via its NLS and NES sequences [60,61]. The NTD of TDP-43 exhibits multiple forms of oligomerization that are critical for proper physiological functions [62,63,64,65,66]. The two RRM domains are essential for binding with RNA/DNA molecules [58,67,68,69,70,71]. The CTD of TDP-43 is inherently disordered and of low complexity, participating in protein–protein interaction, liquid–liquid phase separation, or amyloid aggregation [57,72,73,74,75]. Moreover, TDP-43 also harbors many disease-related mutations, which are mostly in the CTD region [76,77,78].

Under physiological conditions, TDP-43 plays important roles in various cellular processes, involved in the regulation of mRNA splicing, stabilization and transport; gene expression and translation; and mRNA and microRNA biogenesis [57,79,80,81,82,83]. Additionally, TDP-43 can undergo phase separation, forming stress granules (SGs) and other membrane-less organelles in response to cellular stress [74,84,85,86,87]. Given the important functions of TDP-43 in RNA and protein homeostasis, it is unsurprising that the structural damages and dysfunction of TDP-43 may easily alter cellular homeostasis and cause severe neurological diseases.

Under pathological conditions, TDP-43 is prone to oligomerization [88,89,90], aggregation [91,92,93], mislocalization to cytoplasm [91,94] and fragmentation, which gives rise to C-terminal fragments (CTFs) [15,95,96,97]. It can also undergo post-translational modification, including ubiquitination [15,16], phosphorylation [95,98,99], SUMOylation [100] and acetylation [101]. All the biomolecular processes of TDP-43 contribute to the TDP-43 pathologies, also referred to as TDP-43 proteinopathies [57,102,103]. In most TDP-43 proteinopathies, cytoplasmic mislocalization and aggregation are considered very important for disease onset and progression [91,93,94,104,105,106]. Meanwhile, TDP-43-positive intranuclear inclusions have also been observed under certain conditions [107,108,109]. The CTFs of TDP-43, such as TDP-35 and TDP-25, are more prone to aggregation and inclusion formation [96,97,110,111]. Notably, the amyloidogenic core (AC) [75,112] and the Q/N-rich region [113,114] in TDP-43 CTFs are mostly aggregation-prone, essential to TDP-43 inclusion formation. At the same time, the disease-linked mutations in TDP-43 can also enhance intrinsic aggregation [78,115,116,117]. Therefore, the cytoplasmic aggregation and nuclear depletion of TDP-43 are likely to greatly impact diverse biological processes [102,118,119,120,121,122,123]. These aberrant processes impacted by TDP-43 proteinopathies include the disruption of mRNA splicing and RNA metabolism [124,125,126], the disturbance of biomolecular condensation [87,127,128,129] and stress granule formation [84,130,131]. The TDP-43 proteinopathies may also result in the impairment of nucleocytoplasmic transport (NCT) [132,133,134] and axonal transport [81,135], induction of the endoplasmic reticulum and oxidative stresses [136,137,138], apoptosis [139,140], dysfunction of mitochondrion [141,142,143] and also deregulation of protein quality control system (such as UPS and autophagy) [144,145]. All of these TDP-43 proteinopathies may ultimately disrupt molecular (protein, RNA) and cellular homeostasis, which is harmful to biological processes.

So far, there is a growing body of evidence highlighting the molecular mechanism of TDP-43 pathogenesis [57,118,119,146]. Overall, based on the evidence obtained, two predominant viewpoints have been currently raised on the patho-mechanism under pathological conditions. The pathological TDP-43 species disrupt the nuclear pore complex and further impair NCT, resulting in the nuclear depletion and cytoplasmic aggregation of TDP-43 [105,133,134,147]. On the other hand, cytoplasmic TDP-43 aggregates can sequester native TDP-43 [148,149,150] and other RNA-binding proteins (RBPs) [150], as well as their relevant RNAs [151,152,153], leading to a critical loss of their normal functions and the imbalance of cellular homeostasis [150,154,155,156,157]. The cytoplasmic accumulation of TDP-43 elicits toxic gain-of-function and consequently enhances the native TDP-43 functionality or alters the deleterious functions [104,158,159,160,161,162]. However, TDP-43 aggregation or inclusion formation exhibits a complicated combination of the loss-of-normal function and gain-of-toxic function, which is important in the pathogenesis of ALS, FTLD and other NDDs [102,119,163,164]. In addition, TDP-43 aggregates also exhibit prion-like cell-to-cell propagation, which can enhance disease progression [103,118,165,166,167,168]. Thus, the aberrant TDP-43 protein has an important impact on its function and cellular homeostasis, which ultimately result in cellular deterioration or neuronal degeneration.

### 2.2. Co-Aggregation of TDP-43 and Its Co-Pathologies

**Figure 1:**
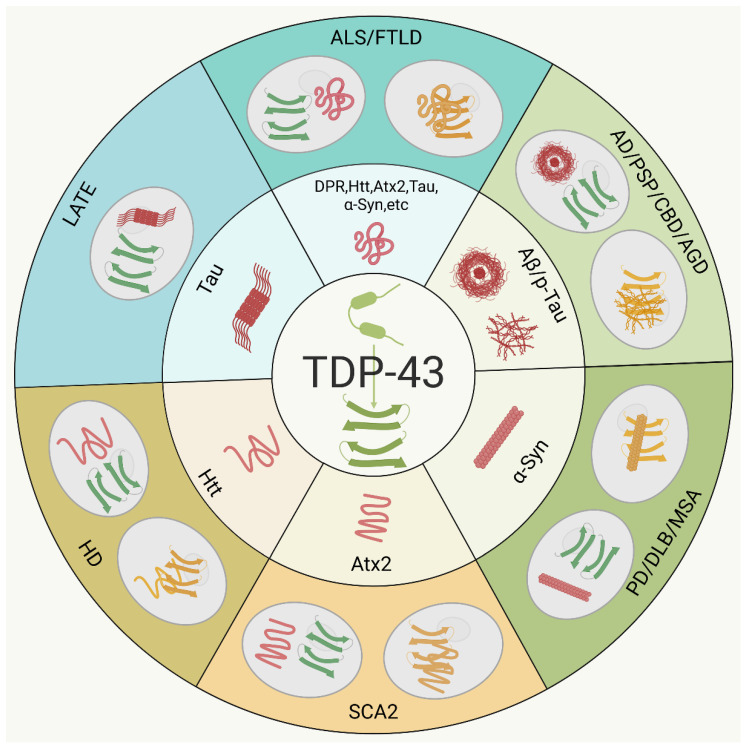
Schematic representation of the co-aggregation of TDP-43 with other pathogenic proteins and their co-pathologies in NDDs. This scheme illustrates the co-aggregation of TDP-43 with other pathogenic proteins and the co-occurrence of their aggregates in inclusions, which become a common feature of most frequently occurring NDDs. The oligomerization or aggregation of TDP-43 (green) is shown in the central circle, while other oligomers or aggregates are depicted in red. The co-occurrence of TDP-43 (green) with other pathogenic proteins (red) in the same neuron or their co-localization in a punctum (yellow) is shown in the outer circles.

To our knowledge, a series of NDDs associated with TDP-43 pathology have been discovered and investigated in detail [59,121,169,170]. In these NDDs, especially ALS and FTLD, the TDP-43 aggregates are generally considered an important component of the pathogenic inclusions. A growing body of evidence reveals the co-occurrence and co-localization of TDP-43 aggregates with other pathogenic proteins in the same neurons, indicating possible interaction and co-aggregation during the protein aggregation processes (Table 1) [22,28,171]. In particular, a subgroup of patients shows the concurrence of more than one TDP-43 pathology-related NDD, and the mixed NDD symptoms exhibit the deposition of TDP-43 in conjunction with other pathogenic proteins in post-mortem brains (Figure 1) [25,50,51,172]. Regardless of the type of NDD, the presence of TDP-43 aggregates directly correlates with the clinical progression. Meanwhile, the co-pathologies of TDP-43 may consequently promote protein aggregation and neurodegeneration, and have an important influence on disease development and progression. Briefly, the TDP-43 aggregates may be a primary cause or a byproduct of the pathological process. From this point of view, we will review and discuss what is known to date about the interaction and co-aggregation between TDP-43 and other pathogenic proteins, as well as the relevance of TDP-43 co-pathologies with disease onset and progression.

## 3. Co-Aggregation of TDP-43 in NDDs

### 3.1. Co-Aggregation of TDP-43 in ALS and FTLD

ALS and FTLD are likely to be multi-factorial neurodegenerative disorders caused by a combination of genetic factors, environmental factors and aging [212,213,214,215]. Cytoplasmic protein aggregates or inclusions are regarded as the well-known pathological hallmark, and there are multiple forms of inclusions described for ALS and FTLD [212,216,217]. The pathological mechanisms underlying ALS and FTLD are elucidated and widely discussed, speculating that they are mediated by complex interaction, co-aggregation and mutual sequestration among the pathogenic proteins, as well as their genetic mutations [23,56,102,119]. To date, the cytoplasmic TDP-43 aggregates, as the most common neuropathological signature and primary causative factor for ALS and FTLD, have been observed in the neurons and glial cells of patients and have been investigated in detail [57,59,102,119,122,169,170,218].

#### 3.1.1. Dipeptide Repeats of C9orf72

Chromosome 9 open reading frame 72 (*C9orf72*) is usually considered one of the most common genetic causes, and the GGGGCC hexanucleotide repeat expansion (referred to as G_4_C_2_ HRE) in *C9orf72* has been found to be associated with ALS and FTTD [219,220,221,222,223]. So far, numerous data suggest that G_4_C_2_ HRE causes ALS and FTLD through both loss-of-function and toxic gain-of-function mechanisms [222,224,225,226,227,228,229]. On one hand, G_4_C_2_ HRE forms G-quadruplex structures and its RNA transcripts accumulate as RNA foci in the cell nuclei [230,231,232,233], which thereby contribute to disrupting biological processes and triggering the progression of ALS and FTLD. On the other hand, G_4_C_2_ HRE can be translated into dipeptide repeats (DPRs, polyGA, polyGP, polyGR, polyPA and polyPR) by repeat-associated non-AUG (RAN) translation [231,234,235], and these five DPRs form aggregates in the cerebral cortex and cerebellum of patients with different abundance and cytotoxicity profiles [223,224,236,237,238,239,240,241]. These aggregates can impair the key physiological process [224,236,242,243,244,245] and, finally, in the models expressing the DPRs, exhibit the neurodegenerative features of ALS and FTLD [246,247]. Moreover, these G_4_C_2_ HRE RNA transcripts and/or DPR aggregates can interact with and sequester specific RBPs or other key cellular factors, potentially preventing them from executing their normal functions [19,232,244,248,249,250,251,252]. Thus, mutations in the *C9orf72* gene go in most instances along with TDP-43 pathology in ALS and FTLD. Not only the RNA foci but also the DPRs can interact with and sequester TDP-43 in vitro and in vivo, triggering the mislocalization and aggregation of TDP-43 and finally impacting the disease onset and progression [207,222,236,249,253]. For instance, polyGA, far more abundant in patients [223], has been proven to be related to TDP-43 aggregation in different experimental models [208,254,255]. A study shows that the polyGA aggregates induce intracellular aggregation of endogenous and exogenous TDP-43, and the detergent-insoluble fraction from the cells co-expressing polyGA and TDP-43 can seed further TDP-43 aggregation [254]. Similarly, polyGA has also been reported to promote sparse nuclear aggregation of TDP-43 in the C9orf72 diseases in a region-specific manner [208]. Other studies reveal that the cytoplasmic polyGA aggregates impair the NCT process [19,210], and co-aggregate with and sequester TDP-43 to induce aggregation and mislocalization [210]. In addition, the cell-to-cell transmission of the polyGA aggregates promotes TDP-43 aggregation by inhibiting the proteasomes [255]. PolyGR, another DPR of *C9orf72*, forms dendritic-like aggregates in the motor cortex and uniquely co-localizes with phosphorylated TDP-43 (*p*-TDP-43) [207]. The arginine-rich DPRs (polyPR and polyGR) are also particularly cytotoxic; they can interact with and sequester the key regulators of NCT and SG assembly, causing NCT defects and SG dynamics disturbance and thereby inducing TDP-43 aggregation and mislocalization [245,249,253,256]. Thus, mutations in *C9orf72* induce the co-aggregation of its RAN DPRs with TDP-43 and, in turn, enhance the aggregation and mislocalization of TDP-43. In this regard, the DPRs, together with TDP-43, are contributable to the co-pathologies of ALS and FTLD.

#### 3.1.2. PolyQ Expansion: Ataxin-2 and Htt

Ataxin-2 (Atx2), as one of the genetic risk factors, can also be accounted for in the generation and development of ALS and FTLD [257,258,259,260]. Several lines of evidence suggest that polyQ expansion in Atx2 is associated with an increased risk of getting ALS and FTLD, especially for the intermediate-length polyQ expansions (27-33Q) [174,210,257,259,261,262,263]. Thus, it is convincing that polyQ-expanded Atx2 often correlates with TDP-43 pathology in vitro and in vivo [174,264]. Two research groups have discovered that Atx2 interacts with TDP-43 in cells, while Atx2 with an intermediate-length polyQ expansion increases the cytoplasmic accumulation and toxicity of TDP-43 in a fly model [257,265], in terms of more severe retinal degeneration and further reduction of lifespan. Meanwhile, these intermediate polyQ-length species also increase TDP-43 mislocalization in the ALS patient-derived cells [257] and enhance stress-induced sequestration of the phosphorylated C-terminal fragment of TDP-43 in human cells via stimulating stress-induced caspase 3 activation [264]. The interaction of Atx2 with TDP-43 contributes to the formation of cytoplasmic SGs, whereas the pathogenic polyQ-expanded Atx2 impairs the SG formation, which results in co-aggregation with TDP-43 and RNA dysregulation [266]. Moreover, polyQ-expanded Atx2 can aberrantly sequester TDP-43 into ribonucleoprotein (RNP) condensates and pertub their liquid-like properties and motilities along axon [267]. A recent study has revealed that the intermediate polyQ (33Q) Atx2 can cause motor deficits, neuromuscular junction changes and Purkinje cell degeneration, and can trigger robust transcriptomic changes through its interaction with the mutant TDP-43 (M337V) [258], indicating that Atx2 intermediate expansions can uncover and exacerbate several ALS-relevant phenotypes in a mutant TDP-43 ALS background. These findings all suggest that the intermediate-length expansions in Atx2 enhance TDP-43 proteinopathies and worsen the degenerative phenotypes, thereby increasing the susceptibility to ALS or FTLD.

Moreover, Htt-related pathology has also been discovered in ALS and FTLD cases [50,268]. Recently, the pathogenic polyQ expansions of Htt have been diagnosed in patients with frontotemporal dementia (FTD) or ALS, in which the classical TDP-43 pathology is shown along with the Htt aggregates in the frontal cortex from autopsy [176], revealing an etiological relationship between polyQ expansion and FTD or ALS syndrome. However, other studies suggest that the *CAG* repeat in the Htt gene has no association with ALS [269,270]. In this regard, more efforts are needed to elucidate the relationship between TDP-43 and Htt in ALS and FTLD.

#### 3.1.3. Tau, Aβ and α-Syn

Other than what has been mentioned above, Tau pathology has also been discovered in ALS and FTLD subjects [173,180,217], in which the co-occurrence of TDP-43 and Tau aggregates in the same neurons in patients has been identified after autopsy studies [178,179,180,265]. In most cases, the phosphorylated Tau (*p*-Tau) aggregates are marked in the brains of patients [271,272], and the TDP-43 level combined with the ratio of *p*-Tau/*t*-Tau can be used as a specific and sensible index for the diagnosis of the diseases [271]. In an animal model, co-expression of TDP-43 and Tau mutants increases the aggregation of *p*-Tau in the hippocampus and is associated with neurodegeneration behavior in a synergistic manner [273]. Cytoplasmic *p*-TDP-43 and *p*-Tau aggregates have also been detected synchronously in the TDP-43-mutated transgenic mice [274]. All these data suggest that pathological TDP-43 can promote Tau aggregation and pathology.

Aβ pathology also exists in the patients of ALS and FTLD [88,275]. With experimental evidence, TDP-43 oligomers can cross-seed the soluble Aβ or Tau to form Aβ/Tau oligomers via the co-aggregation capacity of TDP-43 with Aβ or Tau [88,173]; while the TDP-43 oligomers are also found to accelerate Tau aggregation and co-localize with Tau in the post-mortem brains of ALS and FTD [88].

The α-Syn aggregates have also been detected in a subgroup of ALS and FTLD patients [177,276,277]. α-Syn pathology is sometimes interpreted along with TDP-43 pathology in ALS cases [177,278], and the co-existence of α-Syn and TDP-43 pathologies has been revealed from the clinical documentation of patients in post-mortem research [175,279], suggesting that α-Syn aggregates may play a significant role in the pathogenesis of ALS. Indeed, a study by Robinson et al. reveals that TDP-43 and α-Syn exert a synergistic action in ALS cases [177]. Very recently, several studies have shown that α-Syn can interact and co-aggregate with TDP-43 [280,281,282]. Meanwhile, α-Syn and TDP-43 can co-localize in the cytoplasm of model cells [282,283], while the heterotypic TDP-43 fibrils exhibiting morphological and distribution differences selectively induce synaptic dysfunction in primary neurons [283]. In particular, evidence from cellular and animal models indicates that α-Syn and the prion-like domain of TDP-43 co-aggregate with each other in a synergistic manner and mutually modulate their aggregation behaviors by forming hybrid fibrils [280,282], which finally generate higher cytotoxicity [280,283]. However, mutations in TDP-43 may destabilize the protein and reduce its binding affinity with α-Syn, shedding light on the functional consequences of the mutations in TDP-43 and the mechanism of co-aggregation involving these two proteins [284]. Therefore, the co-aggregation of TDP-43 with α-Syn may aggravate the neurodegeneration of patients.

In a word, the co-aggregation of TDP-43 with other pathogenic proteins in ALS and FTLD may have significant importance in understanding the patho-mechanism of ALS and FTLD. The TDP-43 pathology may act as the primary cause of these diseases, while the presence of other pathologies induced by TDP-43 co-aggregation is associated with the neuropathological features that may worsen the clinical outcomes of ALS and FTLD patients.

### 3.2. Co-Aggregation of TDP-43 with Tau/Aβ in Tauopathies or Aβ-Related Diseases

Not limited to ALS and FTLD, TDP-43 pathology has also been discovered in Tau- and Aβ-related diseases, such as AD [173,185,200,285,286,287,288,289], corticobasal degeneration (CBD) [191,192,193,194,290,291], progressive supranuclear palsy (PSP) [190,191,292] and argyrophilic grain disease (AGD) [195,196]. These diseases are often defined by the presence of Tau and/or Aβ aggregates.

TDP-43 pathology is frequently characterized and confirmed in AD cases, while TDP-43 aggregates can be detected in all six distinctive stages in AD progression in a stereotypical manner [293,294]. An autopsy-based study has reported distinct molecular patterns of TDP-43 pathology between AD and FTLD-TDP cases, which is associated with their clinical symptoms [295]. In fact, the TDP-43 aggregates have been shown to concur with the Tau/Aβ aggregates in AD patients [185,186,286,296]. They are co-deposited in neurons with neurofibrillary tangles (NTFs) and senile plaques in the same neurons [185,187,188,189,296,297,298].

Meanwhile, several lines of experimental evidence suggest the interaction or co-localization between TDP-43 and Aβ or Tau protein, showing that Aβ or Tau oligomers are able to trigger the oligomerization and aggregation of TDP-43 [173,297,299]. Herman et al. have observed the co-localization of Aβ with TDP-43 and an increase of TDP-43 pathology in AD brains when Aβ_42_ is expressed [297]. The expression of Aβ_42_ facilitates phosphorylation, aggregation and modification of TDP-43 in the AD models, indicating that Aβ triggers the TDP-43 pathology and the link between pathological TDP-43 and AD [297]. Other research also indicates that the Tau oligomers can induce mislocalization of TDP-43 and its accumulation into oligomers and aggregates and co-localize with the TDP-43 oligomers in AD, ALS and FTD brain tissues. It suggests that the Tau oligomers are able to co-aggregate with TDP-43, and the interaction between TDP-43 and Tau plays an important role in these diseases [173]. Furthermore, TDP-43 oligomers can also co-aggregate with and trigger Tau/Aβ aggregation in vitro and co-localize with Tau in the post-mortem brains of AD [173,189]. The TDP-43 aggregates are also associated with higher *p*-Tau burden in the AD brain samples [186,300]. At the same time, several studies suggest that both the over-expression and depletion of TDP-43 result in a reduction in the Aβ plaque burden [298,301,302].

Abnormal TDP-43 may exert influence on Tau aggregation and Aβ metabolism in cell and animal models [298,301,303], as well as in AD with comorbid TDP-43 pathology (AD/TDP) [304]. Two research groups have revealed that TDP-43 aggregation and mislocalization into the cytoplasm may modulate its function in the alternative splicing of Tau exon 10, resulting in overexpression of the four-repeat Tau (4R-Tau) protein [80,305,306]. Similarly, TDP-43 interacts with Aβ, participates in the Aβ clearance and promotes Aβ toxicity by stabilizing the oligomeric state and kinetically delaying fibril maturation [189,297,301]. These findings demonstrate that TDP-43 influences the states of Tau/Aβ, whereas abnormal or oligomeric TDP-43 tends to facilitate pathological Tau/Aβ aggregation.

As is known, TDP-43 aggregation in AD brains occurs after Tau aggregation [293], but TDP-43 can synergize with Tau, enhance Tau aggregation and neurotoxicity and, finally, accelerate progressive neurodegeneration [273,298,307]. Indeed, using a *C. elegans* model with modest pan-neuronal co-expression of Tau and TDP-43, one of the most recent studies reveals the robust accumulation of *p*-Tau, severe neuronal dysfunction, progressive neurodegeneration of specific neuronal subtypes owing to the synergy between Tau and TDP-43 and gene expression changes in response to comorbid Tau and TDP-43 [307]. Other studies corroborate this hypothesis, showing increased *p*-Tau aggregation and neurotoxicity and selective neurodegeneration in the *C. elegans* model co-expressing Tau and TDP-43 [303]. Similarly, TDP-43 can interact and co-localize with the intracellular domain of amyloid precursor protein (APP), which enhances its transactivation as well as APP-mediated P53 transcription and apoptosis in several cell models, indicating that TDP-43 may play a role in AD pathology through its interaction with APP [308].

To date, various neuropathological studies have emphasized the effects of the co-pathologies of comorbid TDP-43 with Tau and/or Aβ [285,309,310]. Importantly, TDP-43 influences the clinical features of AD and correlates with the severity of AD pathology, including worse brain atrophy and severe cognitive decline [189,287,288,311,312,313]. It is worth noting that the cytoplasmic TDP-43 aggregates are associated with AD-type dementia, and the pathologic AD mixed with TDP-43 pathologies is associated with more severe AD-type dementia than AD alone [294,314,315]. In general, TDP-43 can interact and co-aggregate with Tau/Aβ in the same neurons, and vice versa. Meanwhile, TDP-43 can exacerbate the aggregation of Tau/Aβ and aggravate their neurotoxicities and AD pathologies, contributing to the clinical features and progression of AD. In this respect, TDP-43 can be recognized as an integral part in AD pathologies.

Similarly, a subgroup of PSP, CBD and AGD cases also shows the presence of TDP-43 pathology in the brain. In most cases, phosphorylated or truncated TDP-43 is regarded as a component of the cytoplasmic aggregates in these disorders, occasionally co-occurring with the Tau pathology. In the case of PSP, TDP-43 aggregates are prominent not only in the hippocampus but also in the limbic area [190,292]. Particularly in the amygdala, the TDP-43 aggregates often co-localize with the Tau aggregates in neuronal cytoplasmic inclusions (NCIs) and dystrophic neurites, which may be associated with the clinical symptoms [190]. There is a report that TDP-43 is presented as NCIs in the dentate granule cells and entorhinal cortex in patients with CBD, and partially co-localized with Tau-positive threads [194]. Severe TDP-43 pathology in the midbrain tectum is strongly associated with downward gaze palsy in CBD patients. It may aggravate the Tau pathology in the olivopontocerebellar system and finally influence the CBD clinical features, suggesting that CBD patients are more vulnerable to TDP-43 pathology [193]. Recent studies have confirmed the mislocalization and cytoplasmic aggregation of TDP-43 in PSP and CBD cases [191,192]. In the spinal cord, the TDP-43 aggregates contain its C-terminal fragments; they co-exist with the 4R-Tau aggregates within the same neuronal cytoplasm but rarely co-localize with each other [191]. Corroborated with the above viewpoint, in AGD subjects, cytoplasmic TDP-43 aggregates tend to be prominent in cases with severe grain pathology, while the co-localization of *p*-TDP-43 and *p*-Tau has been observed only in part of the NCIs, suggesting that the abnormal aggregation of TDP-43 may be involved in the pathological process and disease progression of AGD [195]. Interestingly, the concurrence of ALS/FTLD-TDP with PSP, CBD or AGD has also been reported in the recent literature [49,316]. Therefore, TDP-43 and Tau may co-exist in a co-pathological manner, but the pathogenic proteins rarely co-localize with each other. Overall, it seems that the TDP-43 aggregates are often localized in the cells at certain distances from the Tau aggregates in PSP, CBD and AGD, but TDP-43 and Tau may have pathogenic and mechanistic links in these diseases. The prevalence of the co-pathologies depends on the Tau pathology, where TDP-43 plays a role in the development of Tau pathology or alongside disease progression.

Apart from what has been mentioned above, TDP-43 pathology has also been identified in old age [317,318], which is termed limbic-predominant age-related TDP-43 encephalopathy (LATE). LATE is clinically associated with an amnestic dementia syndrome that mimics AD-type dementia, and is mainly present in patients over 80 years [181]. In addition, LATE neuropathological change (LATE-NC) exhibits a stereotypical TDP-43 proteinopathy in older adults, co-existing with or without hippocampal sclerosis pathology [52,319]. Truncated or phosphorylated TDP-43 aggregates are deposited in LATE, but the deposition pattern of TDP-43 is distinguished from the observed in ALS and FTLD-TDP [181,320]. LATE-NC has been found in the majority of AD cases, leading to a more severe cognitive decline compared with AD pathology alone [51,52,181,182,184]. Specifically, LATE-NC frequently concurs with the AD neuropathologic change [52,181,182], and the co-morbid AD case with LATE-NC exhibits higher *p*-Tau burdens, suggesting that the presence of TDP-43 pathology worsens tauopathy during the pathogenesis of AD [183,184,300]. Furthermore, a subgroup of CBD and AGD cases also display a LATE-NC type [290,321], and comorbid LATE-NC is crucial in individuals with AGD, exhibiting more widespread temporal atrophy [321].

In a word, the comorbid LATE-NC has synergistic effects on cognition compared to one disease alone, suggesting that TDP-43 may contribute to worsening the Tau pathology, probably by exacerbating Tau aggregation. The presence of TDP-43 aggregates is common in Tau- and Aβ-related diseases, especially in the limbic predominant subtypes (e.g., AD and CBD). The TDP-43 protein can co-aggregate with Tau/Aβ in these diseases, while this co-aggregation correlates with their clinical features and even worsens the clinical progression.

### 3.3. Co-Aggregation of TDP-43 with α-Syn in Synucleinopathy-Related Diseases

As mentioned above, TDP-43 aggregates can also be detected in synucleinopathy-related diseases, such as PD with or without dementia [197,198,199,322], dementia with Lewy bodies (DLB) [197,200,201,289] and multiple-system atrophy (MSA) [202,203], in which the pathological signature inclusions are mainly composed of α-Syn. In the brains of most PD and DLB patients, the TDP-43 aggregates co-exist and co-localize with α-Syn in the cytoplasmic inclusions with different patterns [197,200]. It is noteworthy that the co-occurrence of TDP-43 and α-Syn leads to a more severe α-Syn pathology and enhances the neurodegeneration in vitro and in vivo [199,201,323,324]. Thus, TDP-43 exacerbates dopaminergic neuron loss when it is co-expressed with mutant α-Syn in transgenic mice, suggesting that TDP-43 synergistically interacts with α-Syn and aggravates α-Syn toxicity [323]. In addition, *p*-Tau and TDP-43 may accumulate in the dot-like aggregates in mice injected with α-Syn fibrils but they are rarely co-localized with α-Syn pathology, indicating that the α-Syn aggregates have a synergistic effect on Tau and TDP-43 aggregation [325]. Furthermore, the co-occurrence of the α-Syn and TDP-43 aggregates has been observed in a mouse model and SH-SY5Y cells [26]. The synthetic α-Syn fibrils can cause phosphorylation and aggregation of an NLS-deleted TDP-43 form, while *p*-TDP-43 is partially co-localized with the phosphorylated α-Syn [26]. Meanwhile, TDP-43 has been found to co-aggregate with α-Syn in the G51D *SNCA* mutation cases [326]. Similarly, TDP-43 aggregates can also co-localize with α-Syn in glial cytoplasmic inclusions in MSA [202,203]. These neuropathologic findings reveal the co-pathologies of PD and ALS, as well as the co-existence of α-Syn and TDP-43 [279]. The co-deposition of α-Syn and TDP-43 in the motor neuronal nuclei at the levels of the brainstem and spinal cord might account for the multi-system symptoms in a patient [279].

Thus, the α-Syn aggregates may act as the primary cause of the synucleinopathy-related diseases, while TDP-43 co-aggregates with α-Syn in a synergistic manner, and vice versa. This synergistic association promotes the aggregation of TDP-43 and α-Syn, and ultimately leads to more severe α-Syn pathology and acceleration of the disease progression. 

### 3.4. Co-Aggregation of TDP-43 with polyQ Proteins in polyQ Diseases

TDP-43 pathology has also been observed in polyQ diseases, such as HD [40,204] and SCA2 [205,206], in which misfolding and aggregation of the pathogenic proteins induced by polyQ expansion are regarded as the primary causes of these diseases.

Several studies have demonstrated that *p*-TDP-43 can be detected in the intracellular inclusions of mHtt aggregates in the HD brain, and the mHtt aggregates in turn induce the aggregation and co-localization of *p*-TDP-43, suggesting that TDP-43 may participate in co-aggregation with mHtt in HD [204]. Likewise, the mHtt aggregates interact and co-aggregate with TDP-43 in several cell models [327,328], and these co-aggregates of mHtt and *p*-TDP-43 function as seeds to further promote TDP-43 aggregation [327]. More is known about TDP-43 where mHtt aggregates are shown to interact with and sequester TDP-43 via its binding to the C-terminal Q/N-rich region of TDP-43 [328]. Importantly, this co-aggregation of mHtt with TDP-43 reduces the nuclear partition of TDP-43, owing to the increased cytoplasmic accumulation of TDP-43 in the HD brain and cell models, and consequently causes TDP-43 loss of function in RNA processing [328,329]. One recent study has revealed that the cytoplasmic TDP-43 (without NLS) increases and accumulates in the HD knock-in mouse brain, promotes the transport of Htt mRNA into ribosomes via its binding to the Htt pre-mRNA and, finally, results in the aberrant generation of exon1 Htt protein [197]. However, in other autopsied patients with both HD and sporadic ALS, the TDP-43 aggregates and polyQ-immunoreactive inclusions are in co-existence in the same neurons, but they are rarely co-localized within the same inclusions [172]. Another study has also shown that TDP-43 is mis-localized to the cytoplasm of G3BP1 granule-positive HD cortical neurons but not co-localized with the G3BP1 SGs, which implies that the mHtt aggregates may have a greater impact on the critical roles of TDP-43 in the nucleus [330]. The ablation of the nematode ortholog of TDP-43 results in reduced mHtt toxicities in both worm and mammalian cell models with respect to neurodegeneration and behavioral defects, indicating that TDP-43 may be a general modifier of mHtt aggregation, contributing to cellular toxicity propagated by mHtt [331]. All these findings confirm the close relationships between TDP-43 and mHtt proteins. That is, the mHtt aggregates can interact and co-aggregate with TDP-43, which causes an increase in TDP-43 aggregation and loss of its normal function, while the cytoplasmic TDP-43 aggregates in turn lead to the translocation of mHtt pre-mRNA, which thereby results in the production of toxic Htt protein. These molecular events highlight the critical role of TDP-43 in HD progression.

TDP-43 pathology also occurs in SCA2, but knowledge in this field is rather limited. In SCA2 patients, *p*-TDP-43 aggregates have been observed in cytoplasmic inclusions [205,206]. The *p*-TDP-43 and polyQ protein aggregates sometimes co-exist in the same neuron, but there is actually no co-localization that occurs [206]. However, the *p*-TDP-43 aggregates are widely distributed in the central nervous system (CNS) and generally overlap with the polyQ protein, in which TDP-43 is translocated from the nucleus to the cytoplasm along with the translocation of the polyQ protein in the opposite direction, indicating that nuclear aggregation of the mutant Atx2 affects the intracellular dynamics of TDP-43 [205]. In addition, polyQ-expanded Atx2 can progressively sequester TDP-43 into the cytoplasmic Atx2 aggregates in the spinal motor neurons using a novel SCA2 mouse model [332]. Nevertheless, more studies are required to deeply elucidate the relationship between TDP-43 pathology and SCA2.

Altogether, TDP-43 pathology affects polyQ diseases via a specific pathogenetic pathway. Such an interrelationship between a polyQ protein and TDP-43 might promote the cytotoxicities of the polyQ protein and TDP-43 as well, accounting for the progression of co-pathologies in polyQ disorders.

### 3.5. Co-Aggregation of TDP-43 with DCTN1 in Perry Disease

Perry disease (Perry syndrome) is a special hereditary subset of Parkinson’s syndrome, which correlates with the mutations in the *dynactin* (*DCTN1*) gene, while the gene products of the disease-linked DCTN1 mutants can form cytoplasmic inclusions [333,334]. Actually, TDP-43 aggregates have been identified in Perry disease [335,336]. A recent study confirms that the cytoplasmic TDP-43 aggregates are present in an autopsy case of the syndrome without Lewy bodies or Tau pathology, which may correlate to the functional damage in the medulla and hypothalamus [337]. In a *Drosophila* model, the over-expression of the DCTN1 mutants causes defects in axonal transport and the synaptic activity of central dopaminergic neurons, whereas the reduction of TDP-43 expression alleviates these defects [211], suggesting that the stagnation of axonal transport by DCTN1 mutations may promote TDP-43 aggregation. Another study provides supporting evidence that DCTN1 binds to TDP-43 involved in the NCT process of TDP-43, and the disease-linked DCTN1 mutant increases its interaction with TDP-43, triggering the mislocalization and co-aggregation of TDP-43 in non-neuronal cells and the induced pluripotent stem cell (iPSC)-derived neurons [338]. In this regard, the TDP-43 aggregates appear to act as disease modifiers, and its co-aggregation with the DCTN1 mutant may have a great impact on the clinical features of Perry disease.

## 4. Conclusions and Perspectives

Although disease-specific degeneration is probably relevant to the accumulation of one or two pathogenic proteins, the aggregation of multiple pathogenic proteins can concur and sometimes co-localize in the same neurons, leading to synergistic proteinopathies or co-pathologies. Importantly, co-pathologies significantly increase the risk, lower the threshold and accelerate the progression of diseases. The fundamental concept of protein co-aggregation has also been mentioned as one of the important mechanisms underlying the co-pathologies of NDDs. Analogously, recent studies highlight a role for TDP-43 in neuromuscular physiology and myopathy [339], implying that TDP-43 pathology may be associated with inclusion body myositis, a late-onset acquired inflammatory myopathy [340,341]. Therefore, the TDP-43 pathology is not only involved in various NDDs but is also contributable to other non-CNS diseases, such as myopathy.

**Figure 2:**
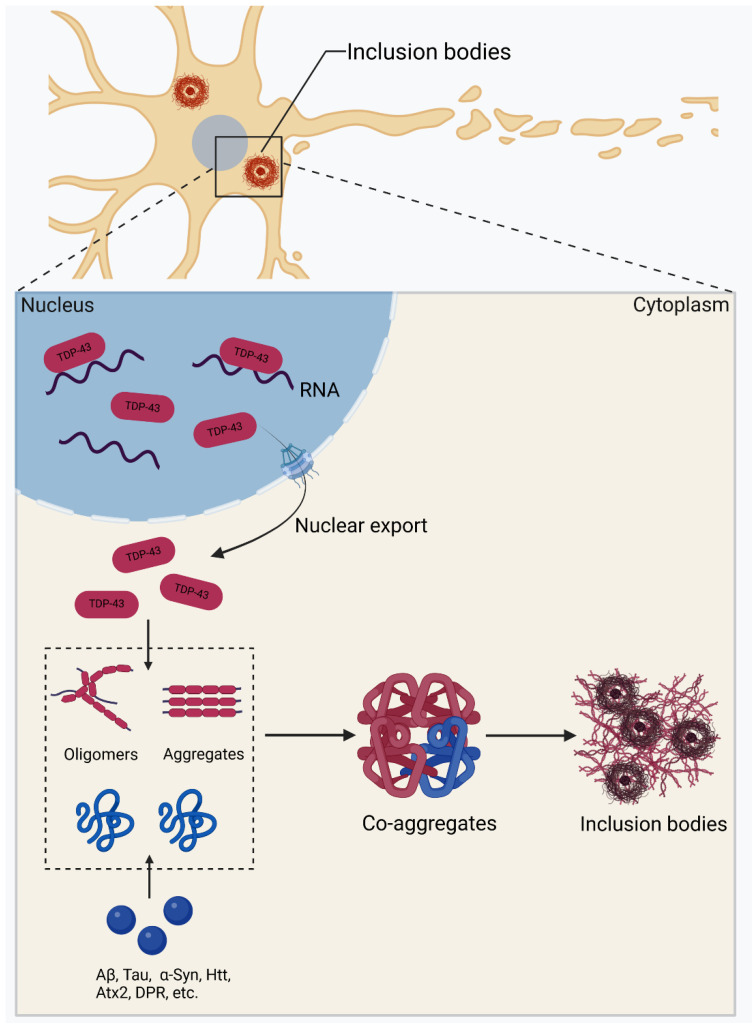
Schematic representation for the co-aggregation process of TDP-43 with other pathogenic proteins. In general, TDP-43 (red) mis-localizes to cytoplasm from the nucleus and forms oligomers or aggregates in the cytoplasm. During the aggregation process, TDP-43 may co-aggregate with other pathogenic proteins (such as Aβ, Tau, α-Syn, Htt, Atx2 and DPR), forming cytoplasmic co-aggregates and finally accumulating in inclusion bodies, which are fundamental to the TDP-43 co-pathology.

In summary, the pathological TDP-43 aggregates deposit in a wide range of NDDs and overlap in the neuropathological processes with other pathogenic proteins. In many cases, abnormal TDP-43 aggregates can trigger the aggregation and spread of other pathogenic proteins, and vice versa. The underlying interaction, co-aggregation and mutual sequestration of TDP-43 with other pathogenic proteins may influence their pathological progression and account for disease development and the clinical manifestation of different NDDs (Figure 2). Furthermore, the molecular species of abnormal TDP-43 are different in diverse diseases, and they propagate from affected cells to other cells during disease progression, which may finally determine the clinicopathological phenotypes. Generally, TDP-43 proteinopathy is regarded as the primary pathology in ALS, FTLD and LATE, and the TDP-43 aggregates may present as the causative factor of these NDDs. Secondly, the TDP-43 proteinopathy can also be regarded as the additional pathology in other NDDs, such as AD, PD, HD and SCA2, in which the TDP-43 aggregates may be a byproduct or a part of the disease process. In these NDDs, the primary pathology frequently affects the prevalence and severity of TDP-43 co-pathology, i.e., the more severe the primary pathology, the higher the incidence of the TDP-43 proteinopathy. Therefore, the awareness of the co-aggregation and the co-existence of TDP-43 with other pathogenic proteins will be beneficial for our understanding of the TDP-43 proteinopathy on disease development and clinical manifestation.

## Figures and Tables

**Table 1:** TDP-43 co-aggregation and its related NDDs.

Predominant Pathogenic Protein	Other Pathogenic Proteins	Pathological Effect	Disease [Reference]	
TDP-43	Aβ, Tau α-Syn Htt, Atx2 *C9orf72* DPRs	Accelerate TDP-43 aggregation; promote Tau aggregation; aggravate neurodegeneration	ALS [173,174,175,176,177] FTLD [88,173,178,179,180]	
TDP-43	Tau	Worsen Tau pathology; lead to more severe cognitive decline	LATE [181,182,183,184]	
Tau and Aβ	TDP-43	Exacerbate Tau/Aβ aggregation; aggravate Tau pathology; increase neurotoxicity; correlate with severe AD pathology	AD [185,186,187,188,189]	
Tau			PSP [190,191,192] CBD [191,193,194] AGD [195,196]	
α-Syn	TDP-43	Promote TDP-43 aggregation; promote α-Syn aggregation; aggravate synucleinopathy	PD [197,198,199] DLB [197,200,201] MSA [202,203]	
Htt	TDP-43	Promote TDP-43 aggregation; perturb TDP-43 function in RNA processing	HD [40,204]	
Atx2	TDP-43	Promote TDP-43 aggregation	SCA2 [205,206]	
*C9orf72* DPRs	TDP-43	Promote TDP-43 aggregation	ALS [207,208,209] FTLD [210]	
DCTN1	TDP-43	Disrupt NCT of TDP-43; promote TDP-43 mislocalization and co-aggregation	Perry disease [211]	

Note: AGD, argyrophilic grain disease; *C9orf72*, chromosome 9 open reading frame 72; CBD, corticobasal degeneration; DCTN1, dynactin; DLB, dementia with Lewy bodies; DPR, dipeptide repeat protein; Htt, huntingtin; LATE, limbic-predominant age-related TDP-43 encephalopathy; MSA, multiple system atrophy; PSP, progressive supranuclear palsy.

## Abbreviation

4R-Tau: four-repeat Tau; AC, amyloidogenic core; AD, Alzheimer’s disease; ADNC, Alzheimer’s disease neuropathologic change; AD/TDP, AD with comorbid TDP-43 pathology; Aβ, amyloid β; AGD, argyrophilic grain disease; ALS, amyotrophic lateral sclerosis; APP, amyloid precursor protein; Atx2, ataxin-2; *C9orf72*, chromosome 9 open reading frame 72; CBD, corticobasal degeneration; CNS, central nervous system; CTD, C-terminal domain; CTF, C-terminal fragment; DCTN1, dynactin; DLB, dementia with Lewy bodies; DPR, dipeptide repeat protein; FTD, frontotemporal dementia; FTLD, frontotemporal lobar degeneration; G3BP1, Ras GTPase-activating protein-binding protein 1; G_4_C_2_ HRE, GGGGCC hexanucleotide repeat expansion; HD, Huntington’s disease; Htt, huntingtin; iPSC, induced pluripotent stem cells; LATE, limbic-predominant age-related TDP-43 encephalopathy; LATE-NC, LATE neuropathological change; MSA, multiple system atrophy; mHtt, mutant huntingtin; NCT, nucleocytoplasmic transport; NDD, neurodegenerative disease; NCI, neuronal cytoplasmic inclusion; NES, nuclear export signal; NLS, nuclear localization signal; NTD, N-terminal domain; NTF, neurofibrillary tangle; PD, Parkinson’s disease; poly-GA, poly (glycine-alanine); poly-GP, poly (glycine-proline); poly-GR, poly (glycine-arginine); poly-PA, poly (proline-alanine); poly-PR, poly (proline-arginine); polyQ, polyglutamine; PrLD, prion-like domain; PSP, progressive supranuclear palsy; *p*-Tau, phosphorylated Tau; *p*-TDP-43, phosphorylated TDP-43; RAN, repeat-associated non-AUG; RBP, RNA-binding protein; RRM, RNA recognition motif; SCA2, spinocerebellar ataxia type 2; SG, stress granule; α-Syn, α-synuclein; Tau, Tau protein; TDP-25, the 25-kDa fragment of TDP-43; TDP-35, the 35-kDa fragment of TDP-43; TDP-43, TAR DNA-binding protein of 43 kDa; WT, wild type.

## References

[1] Candelise N., Scaricamazza S., Salvatori I., Ferri A., Valle C., Manganelli V., Garofalo T., Sorice M., Misasi R. (2021). Protein Aggregation Landscape in Neurodegenerative Diseases: Clinical Relevance and Future Applications. Int. J. Mol. Sci..

[2] Chiti F., Dobson C.M. (2017). Protein Misfolding, Amyloid Formation, and Human Disease: A Summary of Progress Over the Last Decade. Annu. Rev. Biochem..

[3] Tsoi P.S., Quan M.D., Ferreon J.C., Ferreon A.C.M. (2023). Aggregation of Disordered Proteins Associated with Neurodegeneration. Int. J. Mol. Sci..

[4] Ross C.A., Poirier M.A. (2004). Protein aggregation and neurodegenerative disease. Nat. Med..

[5] Wilson D.M., Cookson M.R., Bosch L.V.D., Zetterberg H., Holtzman D.M., Dewachter I. (2023). Hallmarks of neurodegenerative diseases. Cell.

[6] Iadanza M.G., Jackson M.P., Hewitt E.W., Ranson N.A., Radford S.E. (2018). A new era for understanding amyloid structures and disease. Nat. Rev. Mol. Cell Biol..

[7] Morishima-Kawashima M., Ihara Y. (2002). Alzheimer’s disease: Beta-Amyloid protein and tau. J. Neurosci. Res..

[8] Cras P., van Harskamp F., Hendriks L., Ceuterick C., van Duijn C.M., Stefanko S.Z., Hofman A., Kros J.M., Van Broeckhoven C., Martin J.J. (1998). Presenile Alzheimer dementia characterized by amyloid angiopathy and large amyloid core type senile plaques in the APP 692Ala-->Gly mutation. Acta Neuropathol..

[9] Goedert M., Spillantini M.G., Jakes R., Rutherford D., Crowther R.A. (1989). Multiple isoforms of human microtubule-associated protein tau: Sequences and localization in neurofibrillary tangles of Alzheimer’s disease. Neuron.

[10] Lee M.K., Stirling W., Xu Y., Xu X., Qui D., Mandir A.S., Dawson T.M., Copeland N.G., Jenkins N.A., Price D.L. (2002). Human alpha-synuclein-harboring familial Parkinson’s disease-linked Ala-53 --> Thr mutation causes neurodegenerative disease with alpha-synuclein aggregation in transgenic mice. Proc. Natl. Acad. Sci. USA.

[11] Recchia A., Debetto P., Negro A., Guidolin D., Skaper S.D., Giusti P. (2004). Alpha-synuclein and Parkinson’s disease. FASEB J..

[12] Polymeropoulos M.H., Lavedan C., Leroy E., Ide S.E., Dehejia A., Dutra A., Dutra A., Pike B., Root H., Rubenstein J. (1997). Mutation in the alpha-synuclein gene identified in families with Parkinson’s disease. Science.

[13] Saudou F., Humbert S. (2016). The Biology of Huntingtin. Neuron.

[14] Scherzinger E., Sittler A., Schweiger K., Heiser V., Lurz R., Hasenbank R., Bates G.P., Lehrach H., Wanker E.E. (1999). Self-assembly of polyglutamine-containing huntingtin fragments into amyloid-like fibrils: Implications for Huntington’s disease pathology. Proc. Natl. Acad. Sci. USA.

[15] Neumann M., Sampathu D.M., Kwong L.K., Truax A.C., Micsenyi M.C., Chou T.T., Bruce J., Schuck T., Grossman M., Clark C.M. (2006). Ubiquitinated TDP-43 in frontotemporal lobar degeneration and amyotrophic lateral sclerosis. Science.

[16] Arai T., Hasegawa M., Akiyama H., Ikeda K., Nonaka T., Mori H., Mann D., Tsuchiya K., Yoshida M., Hashizume Y. (2006). TDP-43 is a component of ubiquitin-positive tau-negative inclusions in frontotemporal lobar degeneration and amyotrophic lateral sclerosis. Biochem. Biophys. Res. Commun..

[17] Park S.-H., Kukushkin Y., Gupta R., Chen T., Konagai A., Hipp M.S., Hayer-Hartl M., Hartl F.U. (2013). PolyQ proteins interfere with nuclear degradation of cytosolic proteins by sequestering the Sis1p chaperone. Cell.

[18] Engstrom A.K., Walker A.C., Moudgal R.A., Myrick D.A., Kyle S.M., Bai Y., Rowley M.J., Katz D.J. (2020). The inhibition of LSD1 via sequestration contributes to tau-mediated neurodegeneration. Proc. Natl. Acad. Sci. USA.

[19] Zhang Y.-J., Gendron T.F., Grima J.C., Sasaguri H., Jansen-West K., Xu Y.-F., Katzman R.B., Gass J., Murray M.E., Shinohara M. (2016). C9ORF72 poly(GA) aggregates sequester and impair HR23 and nucleocytoplasmic transport proteins. Nat. Neurosci..

[20] Yang H., Hu H. (2016). Sequestration of cellular interacting partners by protein aggregates: Implication in a loss-of-function pathology. FEBS J..

[21] Hu H.-Y., Liu Y.-J. (2022). Sequestration of cellular native factors by biomolecular assemblies: Physiological or pathological?. Biochim. Biophys. Acta Mol. Cell Res..

[22] Moda F., Ciullini A., Dellarole I.L., Lombardo A., Campanella N., Bufano G., Cazzaniga F.A., Giaccone G. (2023). Secondary Protein Aggregates in Neurodegenerative Diseases: Almost the Rule Rather than the Exception. Front. Biosci. Landmark Ed..

[23] Kulichikhin K.Y., Malikova O.A., Zobnina A.E., Zalutskaya N.M., Rubel A.A. (2023). Interaction of Proteins Involved in Neuronal Proteinopathies. Life.

[24] Murakami K., Ono K. (2022). Interactions of amyloid coaggregates with biomolecules and its relevance to neurodegeneration. FASEB J..

[25] Forrest S.L., Kovacs G.G. (2023). Current Concepts of Mixed Pathologies in Neurodegenerative Diseases. Can. J. Neurol. Sci. J. Can. Sci. Neurol..

[26] Nonaka T., Masuda-Suzukake M., Hasegawa M. (2018). Molecular mechanisms of the co-deposition of multiple pathological proteins in neurodegenerative diseases. Neuropathol..

[27] Chaudhuri P., Prajapati K.P., Anand B.G., Dubey K., Kar K. (2019). Amyloid cross-seeding raises new dimensions to understanding of amyloidogenesis mechanism. Ageing Res. Rev..

[28] Calabrese G., Molzahn C., Mayor T. (2022). Protein interaction networks in neurodegenerative diseases: From physiological function to aggregation. J. Biol. Chem..

[29] Badiola N., de Oliveira R.M., Herrera F., Guardia-Laguarta C., Gonçalves S.A., Pera M., Suárez-Calvet M., Clarimon J., Outeiro T.F., Lleó A. (2011). Tau enhances alpha-synuclein aggregation and toxicity in cellular models of synucleinopathy. PLoS ONE.

[30] Roy B., Jackson G.R. (2014). Interactions between Tau and alpha-synuclein augment neurotoxicity in a Drosophila model of Parkinson’s disease. Hum. Mol. Genet..

[31] Bassil F., Meymand E.S., Brown H.J., Xu H., Cox T.O., Pattabhiraman S., Maghames C.M., Wu Q., Zhang B., Trojanowski J.Q. (2021). alpha-Synuclein modulates tau spreading in mouse brains. J. Exp. Med..

[32] Oikawa T., Nonaka T., Terada M., Tamaoka A., Hisanaga S.-I., Hasegawa M. (2016). alpha-Synuclein Fibrils Exhibit Gain of Toxic Function, Promoting Tau Aggregation and Inhibiting Microtubule Assembly. J. Biol. Chem..

[33] Bhasne K., Mukhopadhyay S. (2018). Formation of Heterotypic Amyloids: Alpha-Synuclein in Co-Aggregation. Proteomics.

[34] Clinton L.K., Blurton-Jones M., Myczek K., Trojanowski J.Q., LaFerla F.M. (2010). Synergistic Interactions between Abeta, tau, and alpha-synuclein: Acceleration of neuropathology and cognitive decline. J. Neurosci..

[35] Colom-Cadena M., Gelpi E., Charif S., Belbin O., Blesa R., Martí M.J., Clarimon J., Lleó A. (2013). Confluence of alpha-synuclein, tau, and beta-amyloid pathologies in dementia with Lewy bodies. J. Neuropathol. Exp. Neurol..

[36] Irwin D.J., Hurtig H.I. (2018). The Contribution of Tau, Amyloid-Beta and Alpha-Synuclein Pathology to Dementia in Lewy Body Disorders. J. Alzheimer’s Dis. Park..

[37] Poças G.M., Branco-Santos J., Herrera F., Outeiro T.F., Domingos P.M. (2015). alpha-Synuclein modifies mutant huntingtin aggregation and neurotoxicity in Drosophila. Hum. Mol. Genet..

[38] Charles V., Mezey E., Reddy P., Dehejia A., Young T.A., Polymeropoulos M.H., Brownstein M.J., Tagle D.A. (2000). Alpha-synuclein immunoreactivity of huntingtin polyglutamine aggregates in striatum and cortex of Huntington’s disease patients and transgenic mouse models. Neurosci. Lett..

[39] Furlong R.A., Narain Y., Rankin J., Wyttenbach A., Rubinsztein D.C. (2000). Alpha-synuclein overexpression promotes aggregation of mutant huntingtin. Biochem. J..

[40] St-Amour I., Turgeon A., Goupil C., Planel E., Hébert S.S. (2018). Co-occurrence of mixed proteinopathies in late-stage Huntington’s disease. Acta Neuropathol..

[41] Herrera F., Outeiro T.F. (2012). alpha-Synuclein modifies huntingtin aggregation in living cells. FEBS Lett..

[42] Vuono R., Winder-Rhodes S., de Silva R., Cisbani G., Drouin-Ouellet J., Spillantini M.G., Cicchetti F., Barker R.A., REGISTRY Investigators of the European Huntington’s Disease Network (2015). The role of tau in the pathological process and clinical expression of Huntington’s disease. Brain J. Neurol..

[43] Blum D., Herrera F., Francelle L., Mendes T., Basquin M., Obriot H., Demeyer D., Sergeant N., Gerhardt E., Brouillet E. (2015). Mutant huntingtin alters Tau phosphorylation and subcellular distribution. Hum. Mol. Genet..

[44] Salem S., Cicchetti F. (2023). Untangling the Role of Tau in Huntington’s Disease Pathology. J. Huntingt. Dis..

[45] Salem S., Kilgore M.D., Anwer M., Maxan A., Child D., Bird T.D., Keene C.D., Cicchetti F., Latimer C. (2024). Evidence of mutant huntingtin and tau-related pathology within neuronal grafts in Huntington’s disease cases. Neurobiol. Dis..

[46] Hong J.-Y., Wang J.-Y., Yue H.-W., Zhang X.-L., Zhang S.-X., Jiang L.-L., Hu H.-Y. (2023). Coaggregation of polyglutamine (polyQ) proteins is mediated by polyQ-tract interactions and impairs cellular proteostasis. Acta Biochim. Biophys. Sin..

[47] Bak D., Milewski M. (2010). The composition of the polyglutamine-containing proteins influences their co-aggregation properties. Cell Biol. Int..

[48] Yang J., Xu H., Zhang C., Yang X., Cai W., Chen X. (2023). A prion-like domain of TFEB mediates the co-aggregation of TFEB and mHTT. Autophagy.

[49] Fujita K., Matsubara T., Miyamoto R., Sumikura H., Takeuchi T., Saladini K.M., Kawarai T., Nodera H., Udaka F., Kume K. (2019). Co-morbidity of progressive supranuclear palsy and amyotrophic lateral sclerosis: A clinical-pathological case report. BMC Neurol..

[50] Zhang A.M., Xu H., Huang J.M., Gong H.M., Guo S.M., Lei X.M., He D. (2022). Coexisting amyotrophic lateral sclerosis and chorea: A case report and literature review. Medicine.

[51] Hiya S., Maldonado-Díaz C., Walker J.M., Richardson T.E. (2023). Cognitive symptoms progress with limbic-predominant age-related TDP-43 encephalopathy stage and co-occurrence with Alzheimer disease. J. Neuropathol. Exp. Neurol..

[52] Nelson P.T., Brayne C., Flanagan M.E., Abner E.L., Agrawal S., Attems J., Castellani R.J., Corrada M.M., Cykowski M.D., Di J. (2022). Frequency of LATE neuropathologic change across the spectrum of Alzheimer’s disease neuropathology: Combined data from 13 community-based or population-based autopsy cohorts. Acta Neuropathol..

[53] Pearce M.M. (2017). Prion-like transmission of pathogenic protein aggregates in genetic models of neurodegenerative disease. Curr. Opin. Genet. Dev..

[54] Peng C., Trojanowski J.Q., Lee V.M. (2020). Protein transmission in neurodegenerative disease. Nat. Rev. Neurol..

[55] Davis A.A., Leyns C.E.G., Holtzman D.M. (2018). Intercellular Spread of Protein Aggregates in Neurodegenerative Disease. Annu. Rev. Cell Dev. Biol..

[56] Spires-Jones T.L., Attems J., Thal D.R. (2017). Interactions of pathological proteins in neurodegenerative diseases. Acta Neuropathol..

[57] Prasad A., Bharathi V., Sivalingam V., Girdhar A., Patel B.K. (2019). Molecular Mechanisms of TDP-43 Misfolding and Pathology in Amyotrophic Lateral Sclerosis. Front. Mol. Neurosci..

[58] Ayala Y.M., Pantano S., D’Ambrogio A., Buratti E., Brindisi A., Marchetti C., Romano M., Baralle F.E. (2005). Human, Drosophila, and C.elegans TDP43: Nucleic acid binding properties and splicing regulatory function. J. Mol. Biol..

[59] De Boer E.M.J., Orie V.K., Williams T., Baker M.R., De Oliveira H.M., Polvikoski T., Silsby M., Menon P., van den Bos M., Halliday G.M. (2020). TDP-43 proteinopathies: A new wave of neurodegenerative diseases. J. Neurol. Neurosurg. Psychiatry.

[60] Pinarbasi E.S., Cağatay T., Fung H.Y.J., Li Y.C., Chook Y.M., Thomas P.J. (2018). Active nuclear import and passive nuclear export are the primary determinants of TDP-43 localization. Sci. Rep..

[61] Ayala Y.M., Zago P., D’Ambrogio A., Xu Y.-F., Petrucelli L., Buratti E., Baralle F.E. (2008). Structural determinants of the cellular localization and shuttling of TDP-43. J. Cell Sci..

[62] Jiang L.-L., Xue W., Hong J.-Y., Zhang J.-T., Li M.-J., Yu S.-N., He J.-H., Hu H.-Y. (2017). The N-terminal dimerization is required for TDP-43 splicing activity. Sci. Rep..

[63] Vivoli-Vega M., Guri P., Chiti F., Bemporad F. (2020). Insight into the Folding and Dimerization Mechanisms of the N-Terminal Domain from Human TDP-43. Int. J. Mol. Sci..

[64] Carter G.C., Hsiung C.-H., Simpson L., Yang H., Zhang X. (2021). N-terminal Domain of TDP43 Enhances Liquid-Liquid Phase Separation of Globular Proteins. J. Mol. Biol..

[65] Chang C.-K., Wu T.-H., Wu C.-Y., Chiang M.-H., Toh E.K.-W., Hsu Y.-C., Lin K.-F., Liao Y.-H., Huang T.-H., Huang J.J.-T. (2012). The N-terminus of TDP-43 promotes its oligomerization and enhances DNA binding affinity. Biochem. Biophys. Res. Commun..

[66] Afroz T., Hock E.-M., Ernst P., Foglieni C., Jambeau M., Gilhespy L.A.B., Laferriere F., Maniecka Z., Plückthun A., Mittl P. (2017). Functional and dynamic polymerization of the ALS-linked protein TDP-43 antagonizes its pathologic aggregation. Nat. Commun..

[67] Buratti E., Baralle F.E. (2001). Characterization and functional implications of the RNA binding properties of nuclear factor TDP-43, a novel splicing regulator of CFTR exon 9. J. Biol. Chem..

[68] Kuo P.-H., Doudeva L.G., Wang Y.-T., Shen C.-K.J., Yuan H.S. (2009). Structural insights into TDP-43 in nucleic-acid binding and domain interactions. Nucleic Acids Res..

[69] Kuo P.-H., Chiang C.-H., Wang Y.-T., Doudeva L.G., Yuan H.S. (2014). The crystal structure of TDP-43 RRM1-DNA complex reveals the specific recognition for UG- and TG-rich nucleic acids. Nucleic Acids Res..

[70] Lukavsky P.J., Daujotyte D., Tollervey J.R., Ule J., Stuani C., Buratti E., Baralle F.E., Damberger F.F., Allain F.H.-T. (2013). Molecular basis of UG-rich RNA recognition by the human splicing factor TDP-43. Nat. Struct. Mol. Biol..

[71] Bhardwaj A., Myers M.P., Buratti E., Baralle F.E. (2013). Characterizing TDP-43 interaction with its RNA targets. Nucleic Acids Res..

[72] Chien H.M., Lee C.C., Huang J.J. (2021). The Different Faces of the TDP-43 Low-Complexity Domain: The Formation of Liquid Droplets and Amyloid Fibrils. Int. J. Mol. Sci..

[73] Buratti E., Brindisi A., Giombi M., Tisminetzky S., Ayala Y.M., Baralle F.E. (2005). TDP-43 binds heterogeneous nuclear ribonucleoprotein A/B through its C-terminal tail: An important region for the inhibition of cystic fibrosis transmembrane conductance regulator exon 9 splicing. J. Biol. Chem..

[74] Sun Y., Chakrabartty A. (2017). Phase to Phase with TDP-43. Biochemistry.

[75] Jiang L.-L., Che M.-X., Zhao J., Zhou C.-J., Xie M.-Y., Li H.-Y., He J.-H., Hu H.-Y. (2013). Structural transformation of the amyloidogenic core region of TDP-43 protein initiates its aggregation and cytoplasmic inclusion. J. Biol. Chem..

[76] Lagier-Tourenne C., Cleveland D.W. (2009). Rethinking ALS: The FUS about TDP-43. Cell.

[77] Sreedharan J., Blair I.P., Tripathi V.B., Hu X., Vance C., Rogelj B., Ackerley S., Durnall J.C., Williams K.L., Buratti E. (2008). TDP-43 mutations in familial and sporadic amyotrophic lateral sclerosis. Science.

[78] Jiang L.-L., Zhao J., Yin X.-F., He W.-T., Yang H., Che M.-X., Hu H.-Y. (2016). Two mutations G335D and Q343R within the amyloidogenic core region of TDP-43 influence its aggregation and inclusion formation. Sci. Rep..

[79] Buratti E., Baralle F.E. (2010). The multiple roles of TDP-43 in pre-mRNA processing and gene expression regulation. RNA Biol..

[80] Gu J., Wu F., Xu W., Shi J., Hu W., Jin N., Qian W., Wang X., Iqbal K., Gong C.-X. (2017). TDP-43 suppresses tau expression via promoting its mRNA instability. Nucleic Acids Res..

[81] Alami N.H., Smith R.B., Carrasco M.A., Williams L.A., Winborn C.S., Han S.S., Kiskinis E., Winborn B., Freibaum B.D., Kanagaraj A. (2014). Axonal transport of TDP-43 mRNA granules is impaired by ALS-causing mutations. Neuron.

[82] Kawahara Y., Mieda-Sato A. (2012). TDP-43 promotes microRNA biogenesis as a component of the Drosha and Dicer complexes. Proc. Natl. Acad. Sci. USA.

[83] Ayala Y.M., De Conti L., Avendaño-Vázquez S.E., Dhir A., Romano M., D’Ambrogio A., Tollervey J., Ule J., Baralle M., Buratti E. (2011). TDP-43 regulates its mRNA levels through a negative feedback loop. EMBO J..

[84] Besnard-Guerin C. (2020). Cytoplasmic localization of amyotrophic lateral sclerosis-related TDP-43 proteins modulates stress granule formation. Eur. J. Neurosci..

[85] Khalfallah Y., Kuta R., Grasmuck C., Prat A., Durham H.D., Velde C.V. (2018). TDP-43 regulation of stress granule dynamics in neurodegenerative disease-relevant cell types. Sci. Rep..

[86] McDonald K.K., Aulas A., Destroismaisons L., Pickles S., Beleac E., Camu W., Rouleau G.A., Velde C.V. (2011). TAR DNA-binding protein 43 (TDP-43) regulates stress granule dynamics via differential regulation of G3BP and TIA-1. Hum. Mol. Genet..

[87] Carey J.L., Guo L. (2022). Liquid-Liquid Phase Separation of TDP-43 and FUS in Physiology and Pathology of Neurodegenerative Diseases. Front. Mol. Biosci..

[88] Fang Y.-S., Tsai K.-J., Chang Y.-J., Kao P., Woods R., Kuo P.-H., Wu C.-C., Liao J.-Y., Chou S.-C., Lin V. (2014). Full-length TDP-43 forms toxic amyloid oligomers that are present in frontotemporal lobar dementia-TDP patients. Nat. Commun..

[89] French R.L., Grese Z.R., Aligireddy H., Dhavale D.D., Reeb A.N., Kedia N., Kotzbauer P.T., Bieschke J., Ayala Y.M. (2019). Detection of TAR DNA-binding protein 43 (TDP-43) oligomers as initial intermediate species during aggregate formation. J. Biol. Chem..

[90] Kitamura A., Fujimoto A., Kawashima R., Lyu Y., Sasaki K., Hamada Y., Moriya K., Kurata A., Takahashi K., Brielmann R. (2024). Hetero-oligomerization of TDP-43 carboxy-terminal fragments with cellular proteins contributes to proteotoxicity. Commun. Biol..

[91] Winton M.J., Igaz L.M., Wong M.M., Kwong L.K., Trojanowski J.Q., Lee V.M.-Y. (2008). Disturbance of nuclear and cytoplasmic TAR DNA-binding protein (TDP-43) induces disease-like redistribution, sequestration, and aggregate formation. J. Biol. Chem..

[92] van Eersel J., Ke Y.D., Gladbach A., Bi M., Götz J., Kril J.J., Ittner L.M. (2011). Cytoplasmic accumulation and aggregation of TDP-43 upon proteasome inhibition in cultured neurons. PLoS ONE.

[93] Medina D.X., Orr M.E., Oddo S. (2014). Accumulation of C-terminal fragments of transactive response DNA-binding protein 43 leads to synaptic loss and cognitive deficits in human TDP-43 transgenic mice. Neurobiol. Aging.

[94] Suk T.R., Rousseaux M.W.C. (2020). The role of TDP-43 mislocalization in amyotrophic lateral sclerosis. Mol. Neurodegener..

[95] Arai T., Hasegawa M., Nonoka T., Kametani F., Yamashita M., Hosokawa M., Niizato K., Tsuchiya K., Kobayashi Z., Ikeda K. (2010). Phosphorylated and cleaved TDP-43 in ALS, FTLD and other neurodegenerative disorders and in cellular models of TDP-43 proteinopathy. Neuropathol..

[96] Chhangani D., Martin-Pena A., Rincon-Limas D.E. (2021). Molecular, functional, and pathological aspects of TDP-43 fragmentation. iScience.

[97] Berning B.A., Walker A.K. (2019). The Pathobiology of TDP-43 C-Terminal Fragments in ALS and FTLD. Front. Neurosci..

[98] Hasegawa M., Arai T., Nonaka T., Kametani F., Yoshida M., Hashizume Y., Beach T.G., Buratti E., Baralle F., Morita M. (2008). Phosphorylated TDP-43 in frontotemporal lobar degeneration and amyotrophic lateral sclerosis. Ann. Neurol..

[99] Huang Z., Ba Z., Huang N., Li Y., Luo Y. (2021). Aberrant TDP-43 phosphorylation: A key wind gap from TDP-43 to TDP-43 proteinopathy. Ibrain.

[100] Maraschi A., Gumina V., Dragotto J., Colombrita C., Mompeán M., Buratti E., Silani V., Feligioni M., Ratti A. (2021). SUMOylation Regulates TDP-43 Splicing Activity and Nucleocytoplasmic Distribution. Mol. Neurobiol..

[101] Cohen T.J., Hwang A.W., Restrepo C.R., Yuan C.-X., Trojanowski J.Q., Lee V.M.Y. (2015). An acetylation switch controls TDP-43 function and aggregation propensity. Nat. Commun..

[102] Wood A., Gurfinkel Y., Polain N., Lamont W., Rea S.L. (2021). Molecular Mechanisms Underlying TDP-43 Pathology in Cellular and Animal Models of ALS and FTLD. Int. J. Mol. Sci..

[103] Doke A.A., Jha S.K. (2023). Shapeshifter TDP-43: Molecular mechanism of structural polymorphism, aggregation, phase separation and their modulators. Biophys. Chem..

[104] Barmada S.J., Skibinski G., Korb E., Rao E.J., Wu J.Y., Finkbeiner S. (2010). Cytoplasmic mislocalization of TDP-43 is toxic to neurons and enhanced by a mutation associated with familial amyotrophic lateral sclerosis. J. Neurosci..

[105] Tziortzouda P., Van Den Bosch L., Hirth F. (2021). Triad of TDP43 control in neurodegeneration: Autoregulation, localization and aggregation. Nat. Rev. Neurosci..

[106] Hu Y., Hruscha A., Pan C., Schifferer M., Schmidt M.K., Nuscher B., Giera M., Kostidis S., Burhan Ö., van Bebber F. (2024). Mis-localization of endogenous TDP-43 leads to ALS-like early-stage metabolic dysfunction and progressive motor deficits. Mol. Neurodegener..

[107] Droppelmann C.A., Campos-Melo D., Moszczynski A.J., Amzil H., Strong M.J. (2019). TDP-43 aggregation inside micronuclei reveals a potential mechanism for protein inclusion formation in ALS. Sci. Rep..

[108] Udan-Johns M., Bengoechea R., Bell S., Shao J., Diamond M.I., True H.L., Weihl C.C., Baloh R.H. (2014). Prion-like nuclear aggregation of TDP-43 during heat shock is regulated by HSP40/70 chaperones. Hum. Mol. Genet..

[109] Huang W.-P., Ellis B.C., Hodgson R.E., Avila A.S., Kumar V., Rayment J., Moll T., Shelkovnikova T.A. (2024). Stress-induced TDP-43 nuclear condensation causes splicing loss of function and STMN2 depletion. Cell Rep..

[110] Shenoy J., El Mammeri N., Dutour A., Berbon M., Saad A., Lends A., Morvan E., Grélard A., Lecomte S., Kauffmann B. (2020). Structural dissection of amyloid aggregates of TDP-43 and its C-terminal fragments TDP-35 and TDP-16. FEBS J..

[111] Zhang Y.-J., Xu Y.-F., Cook C., Gendron T.F., Roettges P., Link C.D., Lin W.-L., Tong J., Castanedes-Casey M., Ash P. (2009). Aberrant cleavage of TDP-43 enhances aggregation and cellular toxicity. Proc. Natl. Acad. Sci. USA.

[112] Zhuo X.-F., Wang J., Zhang J., Jiang L.-L., Hu H.-Y., Lu J.-X. (2020). Solid-State NMR Reveals the Structural Transformation of the TDP-43 Amyloidogenic Region upon Fibrillation. J. Am. Chem. Soc..

[113] Budini M., Buratti E., Stuani C., Guarnaccia C., Romano V., De Conti L., Baralle F.E. (2012). Cellular model of TAR DNA-binding protein 43 (TDP-43) aggregation based on its C-terminal Gln/Asn-rich region. J. Biol. Chem..

[114] Sato T., Oda K., Sakai S., Kato R., Yamamori S., Itakura M., Kodera Y., Nishizawa M., Sasaoka T., Onodera O. (2022). Importance of the Q/N-rich segment for protein stability of endogenous mouse TDP-43. Sci. Rep..

[115] Johnson B.S., Snead D., Lee J.J., McCaffery J.M., Shorter J., Gitler A.D. (2009). TDP-43 is intrinsically aggregation-prone, and amyotrophic lateral sclerosis-linked mutations accelerate aggregation and increase toxicity. J. Biol. Chem..

[116] Guo W., Chen Y., Zhou X., Kar A., Ray P., Chen X., Rao E.J., Yang M., Ye H., Zhu L. (2011). An ALS-associated mutation affecting TDP-43 enhances protein aggregation, fibril formation and neurotoxicity. Nat. Struct. Mol. Biol..

[117] Watanabe S., Kaneko K., Yamanaka K. (2013). Accelerated disease onset with stabilized familial amyotrophic lateral sclerosis (ALS)-linked mutant TDP-43 proteins. J. Biol. Chem..

[118] Chen H.J., Mitchell J.C. (2021). Mechanisms of TDP-43 Proteinopathy Onset and Propagation. Int. J. Mol. Sci..

[119] Tamaki Y., Urushitani M. (2022). Molecular Dissection of TDP-43 as a Leading Cause of ALS/FTLD. Int. J. Mol. Sci..

[120] Jiang L., Ngo S.T. (2022). Altered TDP-43 Structure and Function: Key Insights into Aberrant RNA, Mitochondrial, and Cellular and Systemic Metabolism in Amyotrophic Lateral Sclerosis. Metabolites.

[121] Gao J., Wang L., Huntley M.L., Perry G., Wang X. (2018). Pathomechanisms of TDP-43 in neurodegeneration. J. Neurochem..

[122] Klim J.R., Pintacuda G., Nash L.A., Juan I.G.S., Eggan K. (2021). Connecting TDP-43 Pathology with Neuropathy. Trends Neurosci..

[123] Wang X., Hu Y., Xu R. (2024). The pathogenic mechanism of TAR DNA-binding protein 43 (TDP-43) in amyotrophic lateral sclerosis. Neural Regen. Res..

[124] Ling S.C., Polymenidou M., Cleveland D.W. (2013). Converging mechanisms in ALS and FTD: Disrupted RNA and protein homeostasis. Neuron.

[125] Polymenidou M., Lagier-Tourenne C., Hutt K.R., Huelga S.C., Moran J., Liang T.Y., Ling S.-C., Sun E., Wancewicz E., Mazur C. (2011). Long pre-mRNA depletion and RNA missplicing contribute to neuronal vulnerability from loss of TDP-43. Nat. Neurosci..

[126] Brown A.-L., Wilkins O.G., Keuss M.J., Hill S.E., Zanovello M., Lee W.C., Bampton A., Lee F.C.Y., Masino L., Qi Y.A. (2022). TDP-43 loss and ALS-risk SNPs drive mis-splicing and depletion of UNC13A. Nature.

[127] Babinchak W.M., Haider R., Dumm B.K., Sarkar P., Surewicz K., Choi J.-K., Surewicz W.K. (2019). The role of liquid-liquid phase separation in aggregation of the TDP-43 low-complexity domain. J. Biol. Chem..

[128] Conicella A.E., Zerze G.H., Mittal J., Fawzi N.L. (2016). ALS Mutations Disrupt Phase Separation Mediated by alpha-Helical Structure in the TDP-43 Low-Complexity C-Terminal Domain. Structure.

[129] Haider R., Penumutchu S., Boyko S., Surewicz W.K. (2024). Phosphomimetic substitutions in TDP-43’s transiently alpha-helical region suppress phase separation. Biophys. J..

[130] Chen Y., Cohen T.J. (2019). Aggregation of the nucleic acid-binding protein TDP-43 occurs via distinct routes that are coordinated with stress granule formation. J. Biol. Chem..

[131] Aulas A., Vande Velde C. (2015). Alterations in stress granule dynamics driven by TDP-43 and FUS: A link to pathological inclusions in ALS?. Front. Cell. Neurosci..

[132] Woerner A.C., Frottin F., Hornburg D., Feng L.R., Meissner F., Patra M., Tatzelt J., Mann M., Winklhofer K.F., Hartl F.U. (2016). Cytoplasmic protein aggregates interfere with nucleocytoplasmic transport of protein and RNA. Science.

[133] Chou C.-C., Zhang Y., Umoh M.E., Vaughan S.W., Lorenzini I., Liu F., Sayegh M., Donlin-Asp P.G., Chen Y.H., Duong D.M. (2018). TDP-43 pathology disrupts nuclear pore complexes and nucleocytoplasmic transport in ALS/FTD. Nat. Neurosci..

[134] Gasset-Rosa F., Lu S., Yu H., Chen C., Melamed Z., Guo L., Shorter J., Da Cruz S., Cleveland D.W. (2019). Cytoplasmic TDP-43 De-mixing Independent of Stress Granules Drives Inhibition of Nuclear Import, Loss of Nuclear TDP-43, and Cell Death. Neuron.

[135] Sleigh J.N., Tosolini A.P., Gordon D., Devoy A., Fratta P., Fisher E.M., Talbot K., Schiavo G. (2020). Mice Carrying ALS Mutant TDP-43, but Not Mutant FUS, Display In Vivo Defects in Axonal Transport of Signaling Endosomes. Cell Rep..

[136] Lautenschlaeger J., Prell T., Grosskreutz J. (2012). Endoplasmic reticulum stress and the ER mitochondrial calcium cycle in amyotrophic lateral sclerosis. Amyotroph. Lateral Scler..

[137] Dafinca R., Barbagallo P., Talbot K. (2021). The Role of Mitochondrial Dysfunction and ER Stress in TDP-43 and C9ORF72 ALS. Front. Cell. Neurosci..

[138] Pisciottani A., Croci L., Lauria F., Marullo C., Savino E., Ambrosi A., Podini P., Marchioretto M., Casoni F., Cremona O. (2023). Neuronal models of TDP-43 proteinopathy display reduced axonal translation, increased oxidative stress, and defective exocytosis. Front. Cell. Neurosci..

[139] Guerrero E.N., Mitra J., Wang H., Rangaswamy S., Hegde P.M., Basu P., Rao K.S., Hegde M.L. (2019). Amyotrophic lateral sclerosis-associated TDP-43 mutation Q331K prevents nuclear translocation of XRCC4-DNA ligase 4 complex and is linked to genome damage-mediated neuronal apoptosis. Hum. Mol. Genet..

[140] Chhangani D., Rincon-Limas D.E. (2022). TDP-35, a truncated fragment of TDP-43, induces dose-dependent toxicity and apoptosis in flies. Neural Regen. Res..

[141] Gao J., Wang L., Yan T., Perry G., Wang X. (2019). TDP-43 proteinopathy and mitochondrial abnormalities in neurodegeneration. Mol. Cell. Neurosci..

[142] Magrané J., Cortez C., Gan W.-B., Manfredi G. (2014). Abnormal mitochondrial transport and morphology are common pathological denominators in SOD1 and TDP43 ALS mouse models. Hum. Mol. Genet..

[143] Zuo X., Zhou J., Li Y., Wu K., Chen Z., Luo Z., Zhang X., Liang Y., Esteban M.A., Zhou Y. (2021). TDP-43 aggregation induced by oxidative stress causes global mitochondrial imbalance in ALS. Nat. Struct. Mol. Biol..

[144] Budini M., Buratti E., Morselli E., Criollo A. (2017). Autophagy and Its Impact on Neurodegenerative Diseases: New Roles for TDP-43 and C9orf72. Front. Mol. Neurosci..

[145] Riemenschneider H., Guo Q., Bader J., Frottin F., Farny D., Kleinberger G., Haass C., Mann M., Hartl F.U., Baumeister W. (2022). Gel-like inclusions of C-terminal fragments of TDP-43 sequester stalled proteasomes in neurons. EMBO Rep..

[146] Cascella R., Capitini C., Fani G., Dobson C.M., Cecchi C., Chiti F. (2016). Quantification of the Relative Contributions of Loss-of-function and Gain-of-function Mechanisms in TAR DNA-binding Protein 43 (TDP-43) Proteinopathies. J. Biol. Chem..

[147] Nishimura A.L., Župunski V., Troakes C., Kathe C., Fratta P., Howell M., Gallo J., Hortobágyi T., Shaw C.E., Rogelj B. (2010). Nuclear import impairment causes cytoplasmic trans-activation response DNA-binding protein accumulation and is associated with frontotemporal lobar degeneration. Brain J. Neurol..

[148] Keating S.S., Bademosi A.T., Gil R.S., Walker A.K. (2023). Aggregation-prone TDP-43 sequesters and drives pathological transitions of free nuclear TDP-43. Cell. Mol. Life Sci. CMLS.

[149] Che M.-X., Jiang L.-L., Li H.-Y., Jiang Y.-J., Hu H.-Y. (2015). TDP-35 sequesters TDP-43 into cytoplasmic inclusions through binding with RNA. FEBS Lett..

[150] Jiang L.-L., Guan W.-L., Wang J.-Y., Zhang S.-X., Hu H.-Y. (2022). RNA-assisted sequestration of RNA-binding proteins by cytoplasmic inclusions of the C-terminal 35-kDa fragment of TDP-43. J. Cell Sci..

[151] Loganathan S., Lehmkuhl E.M., Eck R.J., Zarnescu D.C. (2019). To Be or Not To Be… Toxic-Is RNA Association With TDP-43 Complexes Deleterious or Protective in Neurodegeneration?. Front. Mol. Biosci..

[152] Louka A., Zacco E., Temussi P.A., Tartaglia G.G., Pastore A. (2020). RNA as the stone guest of protein aggregation. Nucleic Acids Res..

[153] Saldi T.K., Ash P.E., Wilson G., Gonzales P., Garrido-Lecca A., Roberts C.M., Dostal V., Gendron T.F., Stein L.D., Blumenthal T. (2014). TDP-1, the Caenorhabditis elegans ortholog of TDP-43, limits the accumulation of double-stranded RNA. EMBO J..

[154] Liu E.Y., Russ J., Cali C.P., Phan J.M., Amlie-Wolf A., Lee E.B. (2019). Loss of Nuclear TDP-43 Is Associated with Decondensation of LINE Retrotransposons. Cell Rep..

[155] Wood M., Quinet A., Lin Y.-L., Davis A.A., Pasero P., Ayala Y.M., Vindigni A. (2020). TDP-43 dysfunction results in R-loop accumulation and DNA replication defects. J. Cell Sci..

[156] Mitra J., Guerrero E.N., Hegde P.M., Liachko N.F., Wang H., Vasquez V., Gao J., Pandey A., Taylor J.P., Kraemer B.C. (2019). Motor neuron disease-associated loss of nuclear TDP-43 is linked to DNA double-strand break repair defects. Proc. Natl. Acad. Sci. USA.

[157] Yang C., Wang H., Qiao T., Yang B., Aliaga L., Qiu L., Tan W., Salameh J., McKenna-Yasek D.M., Smith T. (2014). Partial loss of TDP-43 function causes phenotypes of amyotrophic lateral sclerosis. Proc. Natl. Acad. Sci. USA.

[158] Charif S.E., Luchelli L., Vila A., Blaustein M., Igaz L.M. (2020). Cytoplasmic Expression of the ALS/FTD-Related Protein TDP-43 Decreases Global Translation Both in vitro and in vivo. Front. Cell. Neurosci..

[159] Lee Y.-B., Scotter E.L., Lee D.-Y., Troakes C., Mitchell J., Rogelj B., Gallo J.-M., Shaw C.E. (2021). Cytoplasmic TDP-43 is involved in cell fate during stress recovery. Hum. Mol. Genet..

[160] Yu C.-H., Davidson S., Harapas C.R., Hilton J.B., Mlodzianoski M.J., Laohamonthonkul P., Louis C., Low R.R.J., Moecking J., De Nardo D. (2020). TDP-43 Triggers Mitochondrial DNA Release via mPTP to Activate cGAS/STING in ALS. Cell.

[161] White M.A., Kim E., Duffy A., Adalbert R., Phillips B.U., Peters O.M., Stephenson J., Yang S., Massenzio F., Lin Z. (2018). TDP-43 gains function due to perturbed autoregulation in a Tardbp knock-in mouse model of ALS-FTD. Nat. Neurosci..

[162] Fratta P., Sivakumar P., Humphrey J., Lo K., Ricketts T., Oliveira H., Brito-Armas J.M., Kalmar B., Ule A., Yu Y. (2018). Mice with endogenous TDP-43 mutations exhibit gain of splicing function and characteristics of amyotrophic lateral sclerosis. EMBO J..

[163] Halliday G., Bigio E.H., Cairns N.J., Neumann M., Mackenzie I.R.A., Mann D.M.A. (2012). Mechanisms of disease in frontotemporal lobar degeneration: Gain of function versus loss of function effects. Acta Neuropathol..

[164] Lee E.B., Lee V.M., Trojanowski J.Q. (2011). Gains or losses: Molecular mechanisms of TDP43-mediated neurodegeneration. Nat. Rev. Neurosci..

[165] Nonaka T., Hasegawa M. (2020). Prion-like properties of assembled TDP-43. Curr. Opin. Neurobiol..

[166] Smethurst P., Newcombe J., Troakes C., Simone R., Chen Y.-R., Patani R., Sidle K. (2016). In vitro prion-like behaviour of TDP-43 in ALS. Neurobiol. Dis..

[167] Porta S., Xu Y., Restrepo C.R., Kwong L.K., Zhang B., Brown H.J., Lee E.B., Trojanowski J.Q., Lee V.M.-Y. (2018). Patient-derived frontotemporal lobar degeneration brain extracts induce formation and spreading of TDP-43 pathology in vivo. Nat. Commun..

[168] Nonaka T., Masuda-Suzukake M., Arai T., Hasegawa Y., Akatsu H., Obi T., Yoshida M., Murayama S., Mann D.M., Akiyama H. (2013). Prion-like properties of pathological TDP-43 aggregates from diseased brains. Cell Rep..

[169] Liao Y.-Z., Ma J., Dou J.-Z. (2022). The Role of TDP-43 in Neurodegenerative Disease. Mol. Neurobiol..

[170] Chen-Plotkin A.S., Lee V.M., Trojanowski J.Q. (2010). TAR DNA-binding protein 43 in neurodegenerative disease. Nat. Rev. Neurol..

[171] Keller B.A., Volkening K., Droppelmann C.A., Ang L.C., Rademakers R., Strong M.J. (2012). Co-aggregation of RNA binding proteins in ALS spinal motor neurons: Evidence of a common pathogenic mechanism. Acta Neuropathol..

[172] Tada M., Coon E.A., Osmand A.P., Kirby P.A., Martin W., Wieler M., Shiga A., Shirasaki H., Tada M., Makifuchi T. (2012). Coexistence of Huntington’s disease and amyotrophic lateral sclerosis: A clinicopathologic study. Acta Neuropathol..

[173] Montalbano M., McAllen S., Cascio F.L., Sengupta U., Garcia S., Bhatt N., Ellsworth A., Heidelman E.A., Johnson O.D., Doskocil S. (2020). TDP-43 and Tau Oligomers in Alzheimer’s Disease, Amyotrophic Lateral Sclerosis, and Frontotemporal Dementia. Neurobiol. Dis..

[174] Hart M.P., Brettschneider J., Lee V.M.Y., Trojanowski J.Q., Gitler A.D. (2012). Distinct TDP-43 pathology in ALS patients with ataxin 2 intermediate-length polyQ expansions. Acta Neuropathol..

[175] Yamada T., Itoh K., Matsuo K., Yamamoto Y., Hosokawa Y., Koizumi T., Shiga K., Mizuno T., Nakagawa M., Fushiki S. (2014). Concomitant alpha-synuclein pathology in an autopsy case of amyotrophic lateral sclerosis presenting with orthostatic hypotension and cardiac arrests. Neuropathology.

[176] Dewan R., Chia R., Ding J., Hickman R.A., Stein T.D., Abramzon Y., Ahmed S., Sabir M.S., Portley M.K., Tucci A. (2021). Pathogenic Huntingtin Repeat Expansions in Patients with Frontotemporal Dementia and Amyotrophic Lateral Sclerosis. Neuron.

[177] Robinson J.L., Lee E.B., Xie S.X., Rennert L., Suh E., Bredenberg C., Caswell C., Van Deerlin V.M., Yan N., Yousef A. (2018). Neurodegenerative disease concomitant proteinopathies are prevalent, age-related and APOE4-associated. Brain J. Neurol..

[178] King A., Al-Sarraj S., Troakes C., Smith B.N., Maekawa S., Iovino M., Spillantini M.G., Shaw C.E. (2013). Mixed tau, TDP-43 and p62 pathology in FTLD associated with a C9ORF72 repeat expansion and p.Ala239Thr MAPT (tau) variant. Acta Neuropathol..

[179] Kim E.-J., Brown J.A., Deng J., Hwang J.-H.L., Spina S., Miller Z.A., DeMay M.G., Valcour V., Karydas A., Ramos E.M. (2018). Mixed TDP-43 proteinopathy and tauopathy in frontotemporal lobar degeneration: Nine case series. J. Neurol..

[180] Takeda T. (2018). Possible concurrence of TDP-43, tau and other proteins in amyotrophic lateral sclerosis/frontotemporal lobar degeneration. Neuropathol..

[181] Nelson P.T., Dickson D.W., Trojanowski J.Q., Jack C.R., Boyle P.A., Arfanakis K., Rademakers R., Alafuzoff I., Attems J., Brayne C. (2019). Limbic-predominant age-related TDP-43 encephalopathy (LATE): Consensus working group report. Brain J. Neurol..

[182] Kapasi A., Yu L., Boyle P.A., Barnes L.L., Bennett D.A., Schneider J.A. (2020). Limbic-predominant age-related TDP-43 encephalopathy, ADNC pathology, and cognitive decline in aging. Neurology.

[183] Latimer C.S., Burke B.T., Liachko N.F., Currey H.N., Kilgore M.D., Gibbons L.E., Henriksen J., Darvas M., Domoto-Reilly K., Jayadev S. (2019). Resistance and resilience to Alzheimer’s disease pathology are associated with reduced cortical pTau and absence of limbic-predominant age-related TDP-43 encephalopathy in a community-based cohort. Acta Neuropathol. Commun..

[184] Kapasi A., Yu L., Leurgans S.E., Agrawal S., Boyle P.A., Bennett D.A., Schneider J.A. (2024). Association between hippocampal microglia, AD and LATE-NC, and cognitive decline in older adults. Alzheimer’s Dement. J. Alzheimer’s Assoc..

[185] Higashi S., Iseki E., Yamamoto R., Minegishi M., Hino H., Fujisawa K., Togo T., Katsuse O., Uchikado H., Furukawa Y. (2007). Concurrence of TDP-43, tau and alpha-synuclein pathology in brains of Alzheimer’s disease and dementia with Lewy bodies. Brain Res..

[186] Llamas-Rodríguez J., Oltmer J., Marshall M., Champion S., Frosch M.P., Augustinack J.C. (2023). TDP-43 and tau concurrence in the entorhinal subfields in primary age-related tauopathy and preclinical Alzheimer’s disease. Brain Pathol..

[187] Carlos A.F., Koga S., Graff-Radford N.R., Baker M.C., Rademakers R., Ross O.A., Dickson D.W., Josephs K.A. (2024). Senile plaque-associated transactive response DNA-binding protein 43 in Alzheimer’s disease: A case report spanning 16 years of memory loss. Neuropathology.

[188] Tomé S.O., Gomes L.A., Li X., Vandenberghe R., Tousseyn T., Thal D.R. (2021). TDP-43 interacts with pathological tau protein in Alzheimer’s disease. Acta Neuropathol..

[189] Shih Y.-H., Tu L.-H., Chang T.-Y., Ganesan K., Chang W.-W., Chang P.-S., Fang Y.-S., Lin Y.-T., Jin L.-W., Chen Y.-R. (2020). TDP-43 interacts with amyloid-beta, inhibits fibrillization, and worsens pathology in a model of Alzheimer’s disease. Nat. Commun..

[190] Yokota O., Davidson Y., Bigio E.H., Ishizu H., Terada S., Arai T., Hasegawa M., Akiyama H., Sikkink S., Pickering-Brown S. (2010). Phosphorylated TDP-43 pathology and hippocampal sclerosis in progressive supranuclear palsy. Acta Neuropathol..

[191] Riku Y., Iwasaki Y., Ishigaki S., Akagi A., Hasegawa M., Nishioka K., Li Y., Riku M., Ikeuchi T., Fujioka Y. (2022). Motor neuron TDP-43 proteinopathy in progressive supranuclear palsy and corticobasal degeneration. Brain J. Neurol..

[192] Robinson J.L., Yan N., Caswell C., Xie S.X., Suh E., Van Deerlin V.M., Gibbons G., Irwin D.J., Grossman M., Lee E.B. (2020). Primary Tau Pathology, Not Copathology, Correlates With Clinical Symptoms in PSP and CBD. J. Neuropathol. Exp. Neurol..

[193] Koga S., Kouri N., Walton R.L., Ebbert M.T.W., Josephs K.A., Litvan I., Graff-Radford N., Ahlskog J.E., Uitti R.J., van Gerpen J.A. (2018). Corticobasal degeneration with TDP-43 pathology presenting with progressive supranuclear palsy syndrome: A distinct clinicopathologic subtype. Acta Neuropathol..

[194] Uryu K., Nakashima-Yasuda H., Forman M.S., Kwong L.K., Clark C.M., Grossman M., Miller B.L., Kretzschmar H.A., Lee V.M.-Y., Trojanowski J.Q. (2008). Concomitant TAR-DNA-binding protein 43 pathology is present in Alzheimer disease and corticobasal degeneration but not in other tauopathies. J. Neuropathol. Exp. Neurol..

[195] Fujishiro H., Uchikado H., Arai T., Hasegawa M., Akiyama H., Yokota O., Tsuchiya K., Togo T., Iseki E., Hirayasu Y. (2009). Accumulation of phosphorylated TDP-43 in brains of patients with argyrophilic grain disease. Acta Neuropathol..

[196] Koga S., Murakami A., Martin N.B., Dickson D.W. (2023). The frequency and distribution of TDP-43 pathology in argyrophilic grain disease. J. Neuropathol. Exp. Neurol..

[197] Kokoulina P., Rohn T.T. (2010). Caspase-cleaved transactivation response DNA-binding protein 43 in Parkinson’s disease and dementia with Lewy bodies. Neuro-Degener. Dis..

[198] Walker L., Attems J. (2024). Prevalence of Concomitant Pathologies in Parkinson’s Disease: Implications for Prognosis, Diagnosis, and Insights into Common Pathogenic Mechanisms. J. Parkinson’s Dis..

[199] Nakashima-Yasuda H., Uryu K., Robinson J., Xie S.X., Hurtig H., Duda J.E., Arnold S.E., Siderowf A., Grossman M., Leverenz J.B. (2007). Co-morbidity of TDP-43 proteinopathy in Lewy body related diseases. Acta Neuropathol..

[200] Arai T., Mackenzie I.R.A., Hasegawa M., Nonoka T., Niizato K., Tsuchiya K., Iritani S., Onaya M., Akiyama H. (2009). Phosphorylated TDP-43 in Alzheimer’s disease and dementia with Lewy bodies. Acta Neuropathol..

[201] Yokota O., Davidson Y., Arai T., Hasegawa M., Akiyama H., Ishizu H., Terada S., Sikkink S., Pickering-Brown S., Mann D.M.A. (2010). Effect of topographical distribution of alpha-synuclein pathology on TDP-43 accumulation in Lewy body disease. Acta Neuropathol..

[202] Koga S., Lin W., Walton R.L., Ross O.A., Dickson D.W. (2018). TDP-43 pathology in multiple system atrophy: Colocalization of TDP-43 and alpha-synuclein in glial cytoplasmic inclusions. Neuropathol. Appl. Neurobiol..

[203] Nwabuobi L., Tomishon D., Shneider N.A., Fahn S., Vonsattel J.P., Cortes E. (2019). Multiple System Atrophy With Predominant Striatonigral Degeneration and TAR DNA-Binding Protein of 43 kDa Pathology: An Unusual Variant of Multiple System Atrophy. Mov. Disord. Clin. Pract..

[204] Schwab C., Arai T., Hasegawa M., Yu S., McGeer P.L. (2008). Colocalization of transactivation-responsive DNA-binding protein 43 and huntingtin in inclusions of Huntington disease. J. Neuropathol. Exp. Neurol..

[205] Koyano S., Yagishita S., Tada M., Doi H., Uchihara T., Tanaka F. (2022). Parallel Appearance of Polyglutamine and Transactivation-Responsive DNA-Binding Protein 43 and Their Complementary Subcellular Localization in Brains of Patients With Spinocerebellar Ataxia Type 2. J. Neuropathol. Exp. Neurol..

[206] Toyoshima Y., Tanaka H., Shimohata M., Kimura K., Morita T., Kakita A., Takahashi H. (2011). Spinocerebellar ataxia type 2 (SCA2) is associated with TDP-43 pathology. Acta Neuropathol..

[207] Saberi S., Stauffer J.E., Jiang J., Garcia S.D., Taylor A.E., Schulte D., Ohkubo T., Schloffman C.L., Maldonado M., Baughn M. (2018). Sense-encoded poly-GR dipeptide repeat proteins correlate to neurodegeneration and uniquely co-localize with TDP-43 in dendrites of repeat-expanded C9orf72 amyotrophic lateral sclerosis. Acta Neuropathol..

[208] LaClair K.D., Zhou Q., Michaelsen M., Wefers B., Brill M.S., Janjic A., Rathkolb B., Farny D., Cygan M., de Angelis M.H. (2020). Congenic expression of poly-GA but not poly-PR in mice triggers selective neuron loss and interferon responses found in C9orf72 ALS. Acta Neuropathol..

[209] Lee Y.-B., Baskaran P., Gomez-Deza J., Chen H.-J., Nishimura A.L., Smith B.N., Troakes C., Adachi Y., Stepto A., Petrucelli L. (2017). C9orf72 poly GA RAN-translated protein plays a key role in amyotrophic lateral sclerosis via aggregation and toxicity. Hum. Mol. Genet..

[210] Khosravi B., Hartmann H., May S., Möhl C., Ederle H., Michaelsen M., Schludi M.H., Dormann D., Edbauer D. (2017). Cytoplasmic poly-GA aggregates impair nuclear import of TDP-43 in C9orf72 ALS/FTLD. Hum. Mol. Genet..

[211] Hosaka Y., Inoshita T., Shiba-Fukushima K., Cui C., Arano T., Imai Y., Hattori N. (2017). Reduced TDP-43 Expression Improves Neuronal Activities in a Drosophila Model of Perry Syndrome. eBioMedicine.

[212] Al-Chalabi A., Hardiman O. (2013). The epidemiology of ALS: A conspiracy of genes, environment and time. Nat. Rev. Neurol..

[213] Koike Y. (2024). Molecular mechanisms linking loss of TDP-43 function to amyotrophic lateral sclerosis/frontotemporal dementia-related genes. Neurosci. Res..

[214] Kim G., Gautier O., Tassoni-Tsuchida E., Ma X.R., Gitler A.D. (2020). ALS Genetics: Gains, Losses, and Implications for Future Therapies. Neuron.

[215] Feldman E.L., Goutman S.A., Petri S., Mazzini L., Savelieff M.G., Shaw P.J., Sobue G. (2022). Amyotrophic lateral sclerosis. Lancet.

[216] Duranti E., Villa C. (2022). Molecular Investigations of Protein Aggregation in the Pathogenesis of Amyotrophic Lateral Sclerosis. Int. J. Mol. Sci..

[217] Hosokawa M., Kondo H., Serrano G.E., Beach T.G., Robinson A.C., Mann D.M., Akiyama H., Hasegawa M., Arai T. (2017). Accumulation of multiple neurodegenerative disease-related proteins in familial frontotemporal lobar degeneration associated with granulin mutation. Sci. Rep..

[218] Cathcart S.J., Appel S.H., Peterson L.E., Greene E.P., Powell S.Z., Arumanayagam A.S., Rivera A.L., Cykowski M.D. (2021). Fast Progression in Amyotrophic Lateral Sclerosis Is Associated With Greater TDP-43 Burden in Spinal Cord. J. Neuropathol. Exp. Neurol..

[219] DeJesus-Hernandez M., Mackenzie I.R., Boeve B.F., Boxer A.L., Baker M., Rutherford N.J., Nicholson A.M., Finch N.A., Flynn H., Adamson J. (2011). Expanded GGGGCC hexanucleotide repeat in noncoding region of C9ORF72 causes chromosome 9p-linked FTD and ALS. Neuron.

[220] Majounie E., Renton A.E., Mok K., Dopper E.G., Waite A., Rollinson S., Chiò A., Restagno G., Nicolaou N., Simon-Sanchez J. (2012). Frequency of the C9orf72 hexanucleotide repeat expansion in patients with amyotrophic lateral sclerosis and frontotemporal dementia: A cross-sectional study. Lancet Neurol..

[221] Renton A.E., Majounie E., Waite A., Simon-Saánchez J., Rollinson S., Gibbs J.R., Schymick J.C., Laaksovirta H., van Swieten J.C., Myllykangas L. (2011). A hexanucleotide repeat expansion in C9ORF72 is the cause of chromosome 9p21-linked ALS-FTD. Neuron.

[222] Mori K., Gotoh S., Uozumi R., Miyamoto T., Akamine S., Kawabe Y., Tagami S., Ikeda M. (2023). RNA Dysmetabolism and Repeat-Associated Non-AUG Translation in Frontotemporal Lobar Degeneration/Amyotrophic Lateral Sclerosis due to C9orf72 Hexanucleotide Repeat Expansion. JMA J..

[223] Mackenzie I.R.A., Frick P., Grässer F.A., Gendron T.F., Petrucelli L., Cashman N.R., Edbauer D., Kremmer E., Prudlo J., Troost D. (2015). Quantitative analysis and clinico-pathological correlations of different dipeptide repeat protein pathologies in C9ORF72 mutation carriers. Acta Neuropathol..

[224] Freibaum B.D., Taylor J.P. (2017). The Role of Dipeptide Repeats in C9ORF72-Related ALS-FTD. Front. Mol. Neurosci..

[225] Gendron T.F., Petrucelli L. (2018). Disease Mechanisms of C9ORF72 Repeat Expansions. Cold Spring Harb. Perspect. Med..

[226] Balendra R., Isaacs A.M. (2018). C9orf72-mediated ALS and FTD: Multiple pathways to disease. Nat. Rev. Neurol..

[227] Jiang J., Zhu Q., Gendron T.F., Saberi S., McAlonis-Downes M., Seelman A., Stauffer J.E., Jafar-Nejad P., Drenner K., Schulte D. (2016). Gain of Toxicity from ALS/FTD-Linked Repeat Expansions in C9ORF72 Is Alleviated by Antisense Oligonucleotides Targeting GGGGCC-Containing RNAs. Neuron.

[228] Zhu Q., Jiang J., Gendron T.F., McAlonis-Downes M., Jiang L., Taylor A., Garcia S.D., Dastidar S.G., Rodriguez M.J., King P. (2020). Reduced C9ORF72 function exacerbates gain of toxicity from ALS/FTD-causing repeat expansion in C9orf72. Nat. Neurosci..

[229] Haeusler A.R., Donnelly C.J., Rothstein J.D. (2016). The expanding biology of the C9orf72 nucleotide repeat expansion in neurodegenerative disease. Nat. Rev. Neurosci..

[230] Lee Y.-B., Chen H.-J., Peres J.N., Gomez-Deza J., Attig J., Štalekar M., Troakes C., Nishimura A.L., Scotter E.L., Vance C. (2013). Hexanucleotide repeats in ALS/FTD form length-dependent RNA foci, sequester RNA binding proteins, and are neurotoxic. Cell Rep..

[231] Zu T., Liu Y., Bañez-Coronel M., Reid T., Pletnikova O., Lewis J., Miller T.M., Harms M.B., Falchook A.E., Subramony S.H. (2013). RAN proteins and RNA foci from antisense transcripts in C9ORF72 ALS and frontotemporal dementia. Proc. Natl. Acad. Sci. USA.

[232] Haeusler A.R., Donnelly C.J., Periz G., Simko E.A., Shaw P.G., Kim M.-S., Maragakis N.J., Troncoso J.C., Pandey A., Sattler R. (2014). C9orf72 nucleotide repeat structures initiate molecular cascades of disease. Nature.

[233] Raguseo F., Wang Y., Li J., Howe M.P., Balendra R., Huyghebaert A., Vadukul D.M., Tanase D.A., Maher T.E., Malouf L. (2023). The ALS/FTD-related C9orf72 hexanucleotide repeat expansion forms RNA condensates through multimolecular G-quadruplexes. Nat. Commun..

[234] Ash P.E.A., Bieniek K.F., Gendron T.F., Caulfield T., Lin W.-L., DeJesus-Hernandez M., van Blitterswijk M.M., Jansen-West K., Paul J.W., Rademakers R. (2013). Unconventional translation of C9ORF72 GGGGCC expansion generates insoluble polypeptides specific to c9FTD/ALS. Neuron.

[235] Gendron T.F., Bieniek K.F., Zhang Y.-J., Jansen-West K., Ash P.E.A., Caulfield T., Daughrity L., Dunmore J.H., Castanedes-Casey M., Chew J. (2013). Antisense transcripts of the expanded C9ORF72 hexanucleotide repeat form nuclear RNA foci and undergo repeat-associated non-ATG translation in c9FTD/ALS. Acta Neuropathol..

[236] Kwon I., Xiang S., Kato M., Wu L., Theodoropoulos P., Wang T., Kim J., Yun J., Xie Y., McKnight S.L. (2014). Poly-dipeptides encoded by the C9orf72 repeats bind nucleoli, impede RNA biogenesis, and kill cells. Science.

[237] Mann D.M., Rollinson S., Robinson A., Callister J.B., Thompson J.C., Snowden J.S., Gendron T., Petrucelli L., Masuda-Suzukake M., Hasegawa M. (2013). Dipeptide repeat proteins are present in the p62 positive inclusions in patients with frontotemporal lobar degeneration and motor neurone disease associated with expansions in C9ORF72. Acta Neuropathol. Commun..

[238] Mori K., Arzberger T., Grässer F.A., Gijselinck I., May S., Rentzsch K., Weng S.-M., Schludi M.H., van der Zee J., Cruts M. (2013). Bidirectional transcripts of the expanded C9orf72 hexanucleotide repeat are translated into aggregating dipeptide repeat proteins. Acta Neuropathol..

[239] Mori K., Weng S.-M., Arzberger T., May S., Rentzsch K., Kremmer E., Schmid B., Kretzschmar H.A., Cruts M., Van Broeckhoven C. (2013). The C9orf72 GGGGCC repeat is translated into aggregating dipeptide-repeat proteins in FTLD/ALS. Science.

[240] Schmitz A., Marques J.P., Oertig I., Maharjan N., Saxena S. (2021). Emerging Perspectives on Dipeptide Repeat Proteins in C9ORF72 ALS/FTD. Front. Cell. Neurosci..

[241] Dedeene L., Van Schoor E., Ospitalieri S., Ronisz A., Weishaupt J.H., Otto M., Ludolph A.C., Scheuerle A., Vandenberghe R., Van Damme P. (2020). Dipeptide repeat protein and TDP-43 pathology along the hypothalamic-pituitary axis in C9orf72 and non-C9orf72 ALS and FTLD-TDP cases. Acta Neuropathol..

[242] Andrade N.S., Ramic M., Esanov R., Liu W., Rybin M.J., Gaidosh G., Abdallah A., Del’olio S., Huff T.C., Chee N.T. (2020). Dipeptide repeat proteins inhibit homology-directed DNA double strand break repair in C9ORF72 ALS/FTD. Mol. Neurodegener..

[243] Pu M., Tai Y., Yuan L., Zhang Y., Guo H., Hao Z., Chen J., Qi X., Wang G., Tao Z. (2022). The contribution of proteasomal impairment to autophagy activation by C9orf72 poly-GA aggregates. Cell. Mol. Life Sci. CMLS.

[244] White M.R., Mitrea D.M., Zhang P., Stanley C.B., Cassidy D.E., Nourse A., Phillips A.H., Tolbert M., Taylor J.P., Kriwacki R.W. (2019). C9orf72 Poly(PR) Dipeptide Repeats Disturb Biomolecular Phase Separation and Disrupt Nucleolar Function. Mol. Cell.

[245] Hayes L.R., Duan L., Bowen K., Kalab P., Rothstein J.D., States U. (2020). C9orf72 arginine-rich dipeptide repeat proteins disrupt karyopherin-mediated nuclear import. eLife.

[246] Milioto C., Carcolé M., Giblin A., Coneys R., Attrebi O., Ahmed M., Harris S.S., Lee B.I., Yang M., Ellingford R.A. (2024). PolyGR and polyPR knock-in mice reveal a conserved neuroprotective extracellular matrix signature in C9orf72 ALS/FTD neurons. Nat. Neurosci..

[247] Xu L., Wang D., Zhao L., Yang Z., Liu X., Li X., Yuan T., Wang Y., Huang T., Bian N. (2023). C9orf72 poly(PR) aggregation in nucleus induces ALS/FTD-related neurodegeneration in cynomolgus monkeys. Neurobiol. Dis..

[248] Zhang Y.-J., Guo L., Gonzales P.K., Gendron T.F., Wu Y., Jansen-West K., O’raw A.D., Pickles S.R., Prudencio M., Carlomagno Y. (2019). Heterochromatin anomalies and double-stranded RNA accumulation underlie C9orf72 poly(PR) toxicity. Science.

[249] Chew J., Cook C., Gendron T.F., Jansen-West K., del Rosso G., Daughrity L.M., Castanedes-Casey M., Kurti A., Stankowski J.N., Disney M.D. (2019). Aberrant deposition of stress granule-resident proteins linked to C9orf72-associated TDP-43 proteinopathy. Mol. Neurodegener..

[250] Zhang K., Donnelly C.J., Haeusler A.R., Grima J.C., Machamer J.B., Steinwald P., Daley E.L., Miller S.J., Cunningham K.M., Vidensky S. (2015). The C9orf72 repeat expansion disrupts nucleocytoplasmic transport. Nature.

[251] Park J., Wu Y., Shao W., Gendron T.F., van der Spek S.J., Sultanakhmetov G., Basu A., Otero P.C., Jones C.J., Jansen-West K. (2023). Poly(GR) interacts with key stress granule factors promoting its assembly into cytoplasmic inclusions. Cell Rep..

[252] Zhu H. (2024). Interference of nuclear speckles: A nexus of RNA foci, dipeptide repeats, and mis-splicing in C9ORF72 ALS/FTD. Neuron.

[253] Cook C.N., Wu Y., Odeh H.M., Gendron T.F., Jansen-West K., del Rosso G., Yue M., Jiang P., Gomes E., Tong J. (2020). C9orf72 poly(GR) aggregation induces TDP-43 proteinopathy. Sci. Transl. Med..

[254] Nonaka T., Masuda-Suzukake M., Hosokawa M., Shimozawa A., Hirai S., Okado H., Hasegawa M. (2018). C9ORF72 dipeptide repeat poly-GA inclusions promote intracellular aggregation of phosphorylated TDP-43. Hum. Mol. Genet..

[255] Khosravi B., LaClair K.D., Riemenschneider H., Zhou Q., Frottin F., Mareljic N., Czuppa M., Farny D., Hartmann H., Michaelsen M. (2020). Cell-to-cell transmission of C9orf72 poly-(Gly-Ala) triggers key features of ALS/FTD. EMBO J..

[256] Hutten S., Usluer S., Bourgeois B., Simonetti F., Odeh H.M., Fare C.M., Czuppa M., Hruska-Plochan M., Hofweber M., Polymenidou M. (2020). Nuclear Import Receptors Directly Bind to Arginine-Rich Dipeptide Repeat Proteins and Suppress Their Pathological Interactions. Cell Rep..

[257] Elden A.C., Kim H.-J., Hart M.P., Chen-Plotkin A.S., Johnson B.S., Fang X., Armakola M., Geser F., Greene R., Lu M.M. (2010). Ataxin-2 intermediate-length polyglutamine expansions are associated with increased risk for ALS. Nature.

[258] Van Damme P., Veldink J.H., van Blitterswijk M., Corveleyn A., van Vught P.W.J., Thijs V., Dubois B., Matthijs G., van den Berg L.H., Robberecht W. (2011). Expanded ATXN2 CAG repeat size in ALS identifies genetic overlap between ALS and SCA2. Neurology.

[259] Sproviero W., Shatunov A., Stahl D., Shoai M., van Rheenen W., Jones A.R., Al-Sarraj S., Andersen P.M., Bonini N.M., Conforti F.L. (2017). ATXN2 trinucleotide repeat length correlates with risk of ALS. Neurobiol. Aging.

[260] Laffita-Mesa J.M., Paucar M., Svenningsson P. (2021). Ataxin-2 gene: A powerful modulator of neurological disorders. Curr. Opin. Neurol..

[261] Ross O.A., Rutherford N.J., Baker M., Soto-Ortolaza A.I., Carrasquillo M.M., DeJesus-Hernandez M., Adamson J., Li M., Volkening K., Finger E. (2011). Ataxin-2 repeat-length variation and neurodegeneration. Hum. Mol. Genet..

[262] Rubino E., Mancini C., Boschi S., Ferrero P., Ferrone M., Bianca S., Zucca M., Orsi L., Pinessi L., Govone F. (2019). ATXN2 intermediate repeat expansions influence the clinical phenotype in frontotemporal dementia. Neurobiol. Aging.

[263] Lattante S., Millecamps S., Stevanin G., Rivaud-Péchoux S., Moigneu C., Camuzat A., Da Barroca S., Mundwiller E., Salachas F., Hannequin D. (2014). Contribution of ATXN2 intermediary polyQ expansions in a spectrum of neurodegenerative disorders. Neurology.

[264] Hart M.P., Gitler A.D. (2012). ALS-associated ataxin 2 polyQ expansions enhance stress-induced caspase 3 activation and increase TDP-43 pathological modifications. J. Neurosci..

[265] Kim H.-J., Raphael A.R., LaDow E.S., McGurk L., Weber R.A., Trojanowski J.Q., Lee V.M.-Y., Finkbeiner S., Gitler A.D., Bonini N.M. (2014). Therapeutic modulation of eIF2alpha phosphorylation rescues TDP-43 toxicity in amyotrophic lateral sclerosis disease models. Nat. Genet..

[266] Nihei Y., Ito D., Suzuki N. (2012). Roles of ataxin-2 in pathological cascades mediated by TAR DNA-binding protein 43 (TDP-43) and Fused in Sarcoma (FUS). J. Biol. Chem..

[267] Wijegunawardana D., Nayak A., Vishal S.S., Venkatesh N., Gopal P.P. (2024). Ataxin-2 polyglutamine expansions aberrantly sequester TDP-43 ribonucleoprotein condensates disrupting mRNA transport and local translation in neurons. Dev. Cell.

[268] Thomas Q., Coarelli G., Heinzmann A., Le Ber I., Amador M.d.M., Durr A. (2021). Questioning the causality of HTT CAG-repeat expansions in FTD/ALS. Neuron.

[269] Ramos E.M., Keagle P., Gillis T., Lowe P., Mysore J.S., Leclerc A.L., Ratti A., Ticozzi N., Gellera C., Gusella J.F. (2012). Prevalence of Huntington’s disease gene CAG repeat alleles in sporadic amyotrophic lateral sclerosis patients. Amyotroph. Lateral Scler..

[270] Lee T., Li Y., Chesi A., Hart M., Ramos D., Jethava N., Hosangadi D., Epstein J., Hodges B., Bonini N. (2011). Evaluating the prevalence of polyglutamine repeat expansions in amyotrophic lateral sclerosis. Neurology.

[271] Bourbouli M., Rentzos M., Bougea A., Zouvelou V., Constantinides V.C., Zaganas I., Evdokimidis I., Kapaki E., Paraskevas G.P. (2017). Cerebrospinal Fluid TAR DNA-Binding Protein 43 Combined with Tau Proteins as a Candidate Biomarker for Amyotrophic Lateral Sclerosis and Frontotemporal Dementia Spectrum Disorders. Dement. Geriatr. Cogn. Disord..

[272] Yang W., Strong M.J. (2012). Widespread neuronal and glial hyperphosphorylated tau deposition in ALS with cognitive impairment. Amyotroph. Lateral Scler..

[273] Moszczynski A.J., Harvey M., Fulcher N., de Oliveira C., McCunn P., Donison N., Bartha R., Schmid S., Strong M.J., Volkening K. (2019). Synergistic toxicity in an in vivo model of neurodegeneration through the co-expression of human TDP-43(M337V) and tau(T175D) protein. Acta Neuropathol. Commun..

[274] Xu Y.-F., Zhang Y.-J., Lin W.-L., Cao X., Stetler C., Dickson D.W., Lewis J., Petrucelli L. (2011). Expression of mutant TDP-43 induces neuronal dysfunction in transgenic mice. Mol. Neurodegener..

[275] Guedes C., Santin R., Costa A.S., Reiter K.C., Hilbig A., Fernandez L.L. (2017). Distinct Phospho-TDP-43 brain distribution in two cases of FTD, one associated with ALS. Dement. Neuropsychol..

[276] Roberts B., Theunissen F., Mastaglia F.L., Akkari P.A., Flynn L.L. (2022). Synucleinopathy in Amyotrophic Lateral Sclerosis: A Potential Avenue for Antisense Therapeutics?. Int. J. Mol. Sci..

[277] Smith R., Hovren H., Bowser R., Bakkar N., Garruto R., Ludolph A., Ravits J., Gaertner L., Murphy D., Lebovitz R. (2024). Misfolded alpha-synuclein in amyotrophic lateral sclerosis: Implications for diagnosis and treatment. Eur. J. Neurol..

[278] Visanji N.P., Lang A.E., Kovacs G.G. (2019). Beyond the synucleinopathies: Alpha synuclein as a driving force in neurodegenerative comorbidities. Transl. Neurodegener..

[279] Oda S., Sano T., Nishikawa N., Mikasa M., Takahashi Y., Takao M. (2021). Amyotrophic lateral sclerosis with muscle weakness and dropped head during the course of Parkinson’s disease: An autopsy case. Rinsho Shinkeigaku = Clin. Neurol..

[280] Dhakal S., Wyant C.E., George H.E., Morgan S.E., Rangachari V. (2021). Prion-like C-Terminal Domain of TDP-43 and alpha-Synuclein Interact Synergistically to Generate Neurotoxic Hybrid Fibrils. J. Mol. Biol..

[281] Jamerlan A.M., An S.S.A. (2022). A Microplate-Based Approach to Map Interactions between TDP-43 and alpha-Synuclein. J. Clin. Med..

[282] Dhakal S., Mondal M., Mirzazadeh A., Banerjee S., Ghosh A., Rangachari V. (2023). alpha-Synuclein emulsifies TDP-43 prion-like domain-RNA liquid droplets to promote heterotypic amyloid fibrils. Commun. Biol..

[283] Dhakal S., Robang A.S., Bhatt N., Puangmalai N., Fung L., Kayed R., Paravastu A.K., Rangachari V. (2022). Distinct neurotoxic TDP-43 fibril polymorphs are generated by heterotypic interactions with alpha-Synuclein. J. Biol. Chem..

[284] Alamri S.H., Haque S., Alghamdi B.S., Tayeb H.O., Azhari S., Farsi R.M., Elmokadem A., Alamri T.A., Harakeh S., Prakash A. (2023). Comprehensive mapping of mutations in TDP-43 and alpha-Synuclein that affect stability and binding. J. Biomol. Struct. Dyn..

[285] Meneses A., Koga S., O’leary J., Dickson D.W., Bu G., Zhao N. (2021). TDP-43 Pathology in Alzheimer’s Disease. Mol. Neurodegener..

[286] Amador-Ortiz C., Lin W.L., Ahmed Z., Personett D., Davies P., Duara R., Graff-Radford N.R., Hutton M.L., Dickson D.W. (2007). TDP-43 immunoreactivity in hippocampal sclerosis and Alzheimer’s disease. Ann. Neurol..

[287] Katsumata Y., Fardo D.W., Kukull W.A., Nelson P.T. (2018). Dichotomous scoring of TDP-43 proteinopathy from specific brain regions in 27 academic research centers: Associations with Alzheimer’s disease and cerebrovascular disease pathologies. Acta Neuropathol. Commun..

[288] Josephs K.A., Dickson D.W., Tosakulwong N., Weigand S.D., Murray M.E., Petrucelli L., Liesinger A.M., Senjem M.L., Spychalla A.J., Knopman D.S. (2017). Rates of hippocampal atrophy and presence of post-mortem TDP-43 in patients with Alzheimer’s disease: A longitudinal retrospective study. Lancet Neurol..

[289] McAleese K.E., Walker L., Erskine D., Thomas A.J., McKeith I.G., Attems J. (2017). TDP-43 pathology in Alzheimer’s disease, dementia with Lewy bodies and ageing. Brain Pathol..

[290] Mimuro M., Iwasaki Y. (2024). Age-Related Pathology in Corticobasal Degeneration. Int. J. Mol. Sci..

[291] Tando S., Kasai T., Mizuta I., Takahashi H., Yaoi T., Saito K., Hojo T., Mizuno T., Hasegawa M., Itoh K. (2021). An autopsy case of corticobasal syndrome due to asymmetric degeneration of the motor cortex and substantia nigra with TDP-43 proteinopathy, associated with Alzheimer’s disease pathology. Neuropathology.

[292] Koga S., Sanchez-Contreras M., Josephs K.A., Uitti R.J., Graff-Radford N., van Gerpen J.A., Cheshire W.P., Wszolek Z.K., Rademakers R., Dickson D.W. (2017). Distribution and characteristics of transactive response DNA binding protein 43 kDa pathology in progressive supranuclear palsy. Mov. Disord..

[293] Josephs K.A., Murray M.E., Whitwell J.L., Tosakulwong N., Weigand S.D., Petrucelli L., Liesinger A.M., Petersen R.C., Parisi J.E., Dickson D.W. (2016). Updated TDP-43 in Alzheimer’s disease staging scheme. Acta Neuropathol..

[294] James B.D., Wilson R.S., Boyle P.A., Trojanowski J.Q., Bennett D.A., Schneider J.A. (2016). TDP-43 stage, mixed pathologies, and clinical Alzheimer’s-type dementia. Brain J. Neurol..

[295] Tomé S.O., Vandenberghe R., Ospitalieri S., Van Schoor E., Tousseyn T., Otto M., von Arnim C.A.F., Thal D.R. (2020). Distinct molecular patterns of TDP-43 pathology in Alzheimer’s disease: Relationship with clinical phenotypes. Acta Neuropathol. Commun..

[296] Smith V.D., Bachstetter A.D., Ighodaro E., Roberts K., Abner E.L., Fardo D.W., Nelson P.T. (2018). Overlapping but distinct TDP-43 and tau pathologic patterns in aged hippocampi. Brain Pathol..

[297] Herman A.M., Khandelwal P.J., Stanczyk B.B., Rebeck G.W., Moussa C.E.-H. (2011). beta-amyloid triggers ALS-associated TDP-43 pathology in AD models. Brain Res..

[298] Davis S.A., Gan K.A., Dowell J.A., Cairns N.J., Gitcho M.A. (2017). TDP-43 expression influences amyloidbeta plaque deposition and tau aggregation. Neurobiol. Dis..

[299] Guerrero-Muñoz M.J., Castillo-Carranza D.L., Krishnamurthy S., Paulucci-Holthauzen A.A., Sengupta U., Lasagna-Reeves C.A., Ahmad Y., Jackson G.R., Kayed R. (2014). Amyloid-beta oligomers as a template for secondary amyloidosis in Alzheimer’s disease. Neurobiol. Dis..

[300] Tomé S.O., Tsaka G., Ronisz A., Ospitalieri S., Gawor K., Gomes L.A., Otto M., von Arnim C.A.F., Van Damme P., Bosch L.V.D. (2023). TDP-43 pathology is associated with increased tau burdens and seeding. Mol. Neurodegener..

[301] Paolicelli R.C., Jawaid A., Henstridge C.M., Valeri A., Merlini M., Robinson J.L., Lee E.B., Rose J., Appel S., Lee V.M.-Y. (2017). TDP-43 Depletion in Microglia Promotes Amyloid Clearance but Also Induces Synapse Loss. Neuron.

[302] LaClair K.D., Donde A., Ling J.P., Jeong Y.H., Chhabra R., Martin L.J., Wong P.C. (2016). Depletion of TDP-43 decreases fibril and plaque beta-amyloid and exacerbates neurodegeneration in an Alzheimer’s mouse model. Acta Neuropathol..

[303] Latimer C.S., Stair J.G., Hincks J.C., Currey H.N., Bird T.D., Keene C.D., Kraemer B.C., Liachko N.F. (2022). TDP-43 promotes tau accumulation and selective neurotoxicity in bigenic Caenorhabditis elegans. Dis. Models Mech..

[304] Minogue G., Kawles A., Zouridakis A., Keszycki R., Macomber A., Lubbat V., Gill N., Mao Q., Flanagan M.E., Zhang H. (2023). Distinct Patterns of Hippocampal Pathology in Alzheimer’s Disease with Transactive Response DNA-binding Protein 43. Ann. Neurol..

[305] Gu J., Chen F., Iqbal K., Gong C.-X., Wang X., Liu F. (2017). Transactive response DNA-binding protein 43 (TDP-43) regulates alternative splicing of tau exon 10: Implications for the pathogenesis of tauopathies. J. Biol. Chem..

[306] Wu R., Zhou D., Shen X., Chen F., Liu F., Gu J. (2021). Phosphorylation of trans-active response DNA-binding protein-of 43 kDa promotes its cytoplasmic aggregation and modulates its function in tau mRNA stability and exon 10 alternative splicing. J. Neurochem..

[307] Jadhav V.S., Stair J.G., Eck R.J., Smukowski S.N., Currey H.N., Toscano L.G., Hincks J.C., Latimer C.S., Valdmanis P.N., Kraemer B.C. (2024). Transcriptomic evaluation of tau and TDP-43 synergism shows tauopathy predominance and reveals potential modulating targets. Neurobiol. Dis..

[308] Wang J., Yan K., Wu Z.-Q., Zheng C.-Y., Xu R.-X., Chen L.-H., Wen Z.-M., Zhao H.-Q., Ma Q.-H. (2014). TDP-43 interaction with the intracellular domain of amyloid precursor protein induces p53-associated apoptosis. Neurosci. Lett..

[309] Riku Y., Yoshida M., Iwasaki Y., Sobue G., Katsuno M., Ishigaki S. (2022). TDP-43 Proteinopathy and Tauopathy: Do They Have Pathomechanistic Links?. Int. J. Mol. Sci..

[310] Chang X.-L., Tan M.-S., Tan L., Yu J.-T. (2016). The Role of TDP-43 in Alzheimer’s Disease. Mol. Neurobiol..

[311] Josephs K.A., Whitwell J.L., Knopman D.S., Hu W.T., Stroh D.A., Baker M., Rademakers R., Boeve B.F., Parisi J.E., Smith G.E. (2008). Abnormal TDP-43 immunoreactivity in AD modifies clinicopathologic and radiologic phenotype. Neurology.

[312] Josephs K.A., Whitwell J.L., Tosakulwong N., Weigand S.D., Murray M.E., Liesinger A.M., Petrucelli L., Senjem M.L., Ivnik R.J., Parisi J.E. (2015). TAR DNA-binding protein 43 and pathological subtype of Alzheimer’s disease impact clinical features. Ann. Neurol..

[313] Josephs K.A., Whitwell J.L., Weigand S.D., Murray M.E., Tosakulwong N., Liesinger A.M., Petrucelli L., Senjem M.L., Knopman D.S., Boeve B.F. (2014). TDP-43 is a key player in the clinical features associated with Alzheimer’s disease. Acta Neuropathol..

[314] Tremblay C., St-Amour I., Schneider J., Bennett D.A., Calon F. (2011). Accumulation of transactive response DNA binding protein 43 in mild cognitive impairment and Alzheimer disease. J. Neuropathol. Exp. Neurol..

[315] Buciuc M., Whitwell J.L., Tosakulwong N., Weigand S.D., Murray M.E., Boeve B.F., Knopman D.S., Parisi J.E., Petersen R.C., Dickson D.W. (2020). Association between transactive response DNA-binding protein of 43 kDa type and cognitive resilience to Alzheimer’s disease: A case-control study. Neurobiol. Aging.

[316] Koga S., Zhou X., Murakami A., De Castro C.F., Baker M.C., Rademakers R., Dickson D.W. (2022). Concurrent tau pathologies in frontotemporal lobar degeneration with TDP-43 pathology. Neuropathol. Appl. Neurobiol..

[317] Wilson R.S., Yu L., Trojanowski J.Q., Chen E.-Y., Boyle P.A., Bennett D.A., Schneider J.A. (2013). TDP-43 pathology, cognitive decline, and dementia in old age. JAMA Neurol..

[318] Uchino A., Takao M., Hatsuta H., Sumikura H., Nakano Y., Nogami A., Saito Y., Arai T., Nishiyama K., Murayama S. (2015). Incidence and extent of TDP-43 accumulation in aging human brain. Acta Neuropathol. Commun..

[319] Tomé S.O., Gawor K., Thal D.R. (2023). LATE-NC in Alzheimer’s disease: Molecular aspects and synergies. Brain Pathol..

[320] Robinson J.L., Porta S., Garrett F.G., Zhang P., Xie S.X., Suh E., Van Deerlin V.M., Abner E.L., Jicha G.A., Barber J.M. (2020). Limbic-predominant age-related TDP-43 encephalopathy differs from frontotemporal lobar degeneration. Brain J. Neurol..

[321] Inui S., Kaneda D., Sakurai K., Morimoto S., Uchida Y., Abe O., Hashizume Y. (2024). The influence of limbic-predominant age-related TDP-43 encephalopathy on argyrophilic grain disease: A voxel-based morphometry analysis of pathologically confirmed cases. J. Neurol. Sci..

[322] Yamashita R., Beck G., Yonenobu Y., Inoue K., Mitsutake A., Ishiura H., Hasegawa M., Murayama S., Mochizuki H. (2022). TDP-43 Proteinopathy Presenting with Typical Symptoms of Parkinson’s Disease. Mov. Disord..

[323] Tian T., Huang C., Tong J., Yang M., Zhou H., Xia X.-G. (2011). TDP-43 potentiates alpha-synuclein toxicity to dopaminergic neurons in transgenic mice. Int. J. Biol. Sci..

[324] Shen L., Wang C., Chen L., Leung K.L., Lo E., Lakso M., Wong G. (2020). TDP-1/TDP-43 potentiates human alpha-Synuclein (HASN) neurodegeneration in Caenorhabditis elegans. Biochim. Biophys. Acta Mol. Basis Dis..

[325] Masuda-Suzukake M., Nonaka T., Hosokawa M., Kubo M., Shimozawa A., Akiyama H., Hasegawa M. (2014). Pathological alpha-synuclein propagates through neural networks. Acta Neuropathol. Commun..

[326] Kiely A.P., Ling H., Asi Y.T., Kara E., Proukakis C., Schapira A.H., Morris H.R., Roberts H.C., Lubbe S., Limousin P. (2015). Distinct clinical and neuropathological features of G51D SNCA mutation cases compared with SNCA duplication and H50Q mutation. Mol. Neurodegener..

[327] Coudert L., Nonaka T., Bernard E., Hasegawa M., Schaeffer L., Leblanc P. (2019). Phosphorylated and aggregated TDP-43 with seeding properties are induced upon mutant Huntingtin (mHtt) polyglutamine expression in human cellular models. Cell. Mol. Life Sci. CMLS.

[328] Fuentealba R.A., Udan M., Bell S., Wegorzewska I., Shao J., Diamond M.I., Weihl C.C., Baloh R.H. (2010). Interaction with polyglutamine aggregates reveals a Q/N-rich domain in TDP-43. J. Biol. Chem..

[329] Nguyen T.B., Miramontes R., Chillon-Marinas C., Maimon R., Vazquez-Sanchez S., Lau A.L., McClure N.R., England W.E., Singha M., Stocksdale J.T. (2023). Aberrant splicing in Huntington’s disease via disrupted TDP-43 activity accompanied by altered m6A RNA modification. bioRxiv.

[330] Sanchez I.I., Nguyen T.B., England W.E., Lim R.G., Vu A.Q., Miramontes R., Byrne L.M., Markmiller S., Lau A.L., Orellana I. (2021). Huntington’s disease mice and human brain tissue exhibit increased G3BP1 granules and TDP43 mislocalization. J. Clin. Investig..

[331] Tauffenberger A., Chitramuthu B.P., Bateman A., Bennett H.P., Parker J.A. (2013). Reduction of polyglutamine toxicity by TDP-43, FUS and progranulin in Huntington’s disease models. Hum. Mol. Genet..

[332] Canet-Pons J., Sen N.-E., Arsović A., Almaguer-Mederos L.-E., Halbach M.V., Key J., Döring C., Kerksiek A., Picchiarelli G., Cassel R. (2021). Atxn2-CAG100-KnockIn mouse spinal cord shows progressive TDP43 pathology associated with cholesterol biosynthesis suppression. Neurobiol. Dis..

[333] Farrer M.J., Hulihan M.M., Kachergus J.M., Dächsel J.C., Stoessl A.J., Grantier L.L., Calne S., Calne D.B., Lechevalier B., Chapon F. (2009). DCTN1 mutations in Perry syndrome. Nat. Genet..

[334] Mishima T., Yuasa-Kawada J., Fujioka S., Tsuboi Y. (2024). Perry Disease: Bench to Bedside Circulation and a Team Approach. Biomedicines.

[335] Wider C., Dickson D.W., Stoessl A.J., Tsuboi Y., Chapon F., Gutmann L., Lechevalier B., Calne D.B., Personett D.A., Hulihan M. (2009). Pallidonigral TDP-43 pathology in Perry syndrome. Park. Relat. Disord..

[336] Mishima T., Koga S., Lin W.-L., Kasanuki K., Castanedes-Casey M., Wszolek Z.K., Oh S.J., Tsuboi Y., Dickson D.W. (2017). Perry Syndrome: A Distinctive Type of TDP-43 Proteinopathy. J. Neuropathol. Exp. Neurol..

[337] Kim D.D., Alghefari H., Jenkins M., Ang L., Pasternak S.H. (2021). Neuropathology of Perry Syndrome: Evidence of Medullary and Hypothalamic Involvement. Mov. Disord. Clin. Pract..

[338] Deshimaru M., Kinoshita-Kawada M., Kubota K., Watanabe T., Tanaka Y., Hirano S., Ishidate F., Hiramoto M., Ishikawa M., Uehara Y. (2021). DCTN1 Binds to TDP-43 and Regulates TDP-43 Aggregation. Int. J. Mol. Sci..

[339] Versluys L., Pereira P.E., Schuermans N., De Paepe B., De Bleecker J.L., Bogaert E., Dermaut B. (2022). Expanding the TDP-43 Proteinopathy Pathway From Neurons to Muscle: Physiological and Pathophysiological Functions. Front. Neurosci..

[340] Văcăraş V., Vulturar R., Chiş A., Damian L. (2024). Inclusion body myositis, viral infections, and TDP-43: A narrative review. Clin. Exp. Med..

[341] Pereira P.E., Schuermans N., Meylemans A., LeBlanc P., Versluys L., Copley K.E., Rubien J.D., Altheimer C., Peetermans M., Debackere E. (2023). C-terminal frameshift variant of TDP-43 with pronounced aggregation-propensity causes rimmed vacuole myopathy but not ALS/FTD. Acta Neuropathol..

